# BMC Ecology Image Competition 2016: the winning images

**DOI:** 10.1186/s12898-016-0090-z

**Published:** 2016-08-09

**Authors:** Julia Simundza, Matthew Palmer, Josef Settele, Luke M. Jacobus, David P. Hughes, Dominique Mazzi, Simon Blanchet

**Affiliations:** 1 BMC Ecology, BioMed Central, 233 Spring Street, New York, NY 10013 USA; 2 Ecology, Evolution, and Environmental Biology Department, Columbia University, 10th Floor Schermerhorn EXT, 1200 Amsterdam Avenue, New York, NY 10027 USA; 3 UFZ Centre for Environmental Research, Theodor-Lieser-Str. 4, 06120 Halle, Germany; 4 Division of Science, Indiana University Purdue University Columbus (IUPUC), 4601 Central Avenue, Columbus, IN 47203 USA; 5 Department of Entomology, Penn State College of Agricultural Sciences, University Park, PA 16802 USA; 6 Federal Department of Economic Affairs, Education and Research EAER, Agroscope, Institute for Plant Production Sciences IPS, Schloss 1, 8820 Wädenswil, Switzerland; 7 Station d’Ecologie Expérimentale du CNRS, 2 route du CNRS, 09200 Moulis, France; 8 German Centre for Integrative Biodiversity Research (iDiv), Halle-Jena-Leipzig, Deutscher Platz 5e, 04103 Leipzig, Germany

## Abstract

The 2016 BMC Ecology Image Competition marked another celebration of the astounding biodiversity, natural beauty, and biological interactions documented by talented ecologists worldwide. For our fourth annual competition, we welcomed guest judge Dr. Matthew Palmer of Columbia University, who chose the winning image from over 140 entries. In this editorial, we highlight the award winning images along with a selection of highly commended honorable mentions.

**Electronic supplementary material:**

The online version of this article (doi:10.1186/s12898-016-0090-z) contains supplementary material, which is available to authorized users.

## Winning images

The 2016 competition yielded an impressive collection of entries spanning many facets of the natural world, from sprawling vistas and cloudscapes to the fine-tuned interactions of predators, prey, parasites and pollinators. While ecological activity constantly surrounds us, these powerful examples were brought into focus by the expert observational and photographic skills of our contestants. As in previous competitions [1–3], we were thrilled to once again receive such an impressive and varied collection of images this year, and we commend everyone who entered for their excellent work that continues to make this competition a success.

**Fig. 1:**
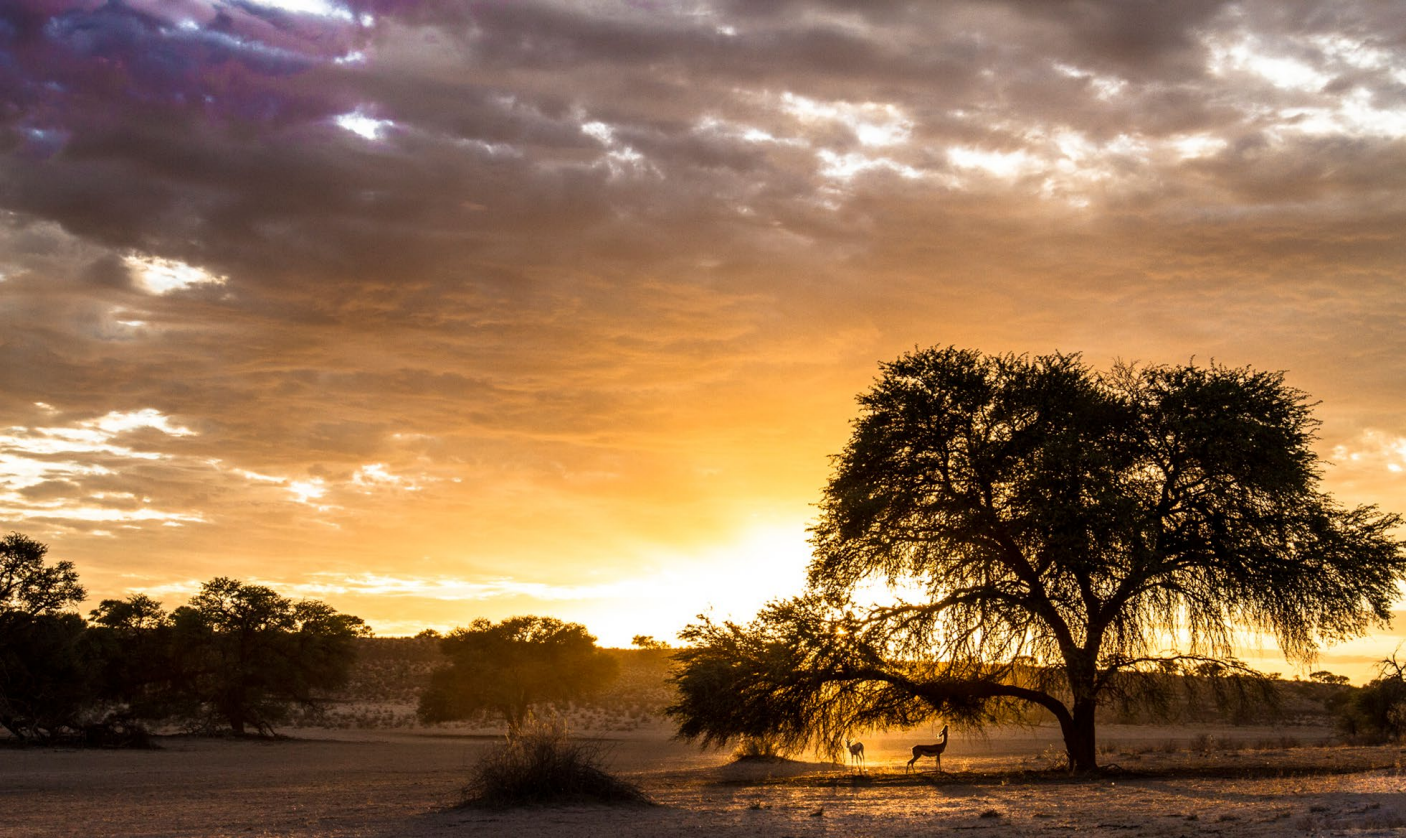
Overall winner: *“The striking landscape of the* *Kgalagadi Transfrontier Park during sunrise. This south African park is characterized by vast arid landscapes with red* *dunes, sparse vegetation and camel thorn trees.”* Attribution: David Giglio

This year’s overall winner deftly encompassed a wide range of ecological interaction within one finely composed photograph. Congratulations to Davide Gaglio of the Percy Fitzpatrick Institute at the University of Cape Town, whose snapshot of the Kalahari Desert (Fig. 1) stood out above all the rest.

Guest judge Matthew Palmer provides a breakdown of the rich and surprising details in Davide’s photo: *“This image is strikingly beautiful*—*particularly the colors and the composition*—*but it also tells several stories. The most obvious story is the antelope browsing on the tree branches*—*probably springbok* (Antidorcas marsupialis)*, though the photographer does not specify. This park is on the edge of the Kalahari Desert in southern Africa, an arid landscape with sparse vegetation. Tree leaves in arid landscapes generally have the greatest water content just before dawn, a fact that is surely not lost on the antelope. A deeper story here is about the park itself. The Kgalagadi Transfrontier Park spans the border of South Africa and Botswana and is an example of cooperation and shared management between countries*—*a peace park. However, large areas of this park were leased for the extraction of natural gas in 2014, which may have negative effects on the park’s wildlife.”*

## Runners-up

**Fig. 2:**
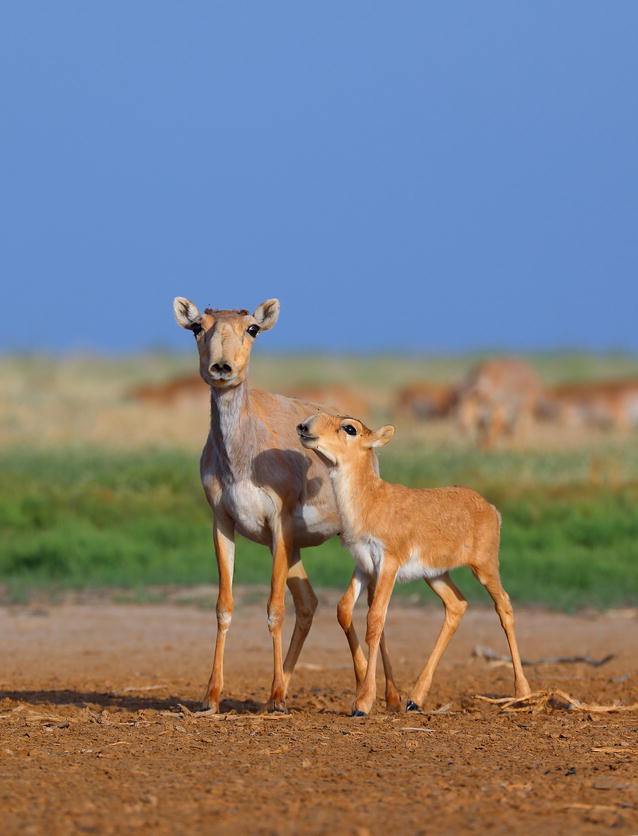
First runner up: *“Saiga antelope is a critically endangered species living in one of the most damaged and fragile ecoregion*—*the Pre*–*Caspian steppe. The newborn saigas which are born every spring are the only hope for the species to survive." *Attribution: Andrey Giljov

Coincidentally, the first runner-up image also features antelope. However unlike the silhouette seen in Davide’s landscape image, the young Saiga antelope are the central subject of the portrait captured by Andrey Giljov of St. Petersburg State University in Russia (Fig. 2). This striking presentation provides an intimate view of the critically endangered species, allowing us to appreciate their unique beauty as well as the importance of conservation efforts.

Matthew Palmer describes the significance of this rare sighting: *“This image highlights two newly*-*born Saiga antelope* (Saiga tatarica) *on the Caspian steppe. The photograph has several wonderful aesthetic elements*—*the comical expressions on the faces of the antelope (largely helped by the enlarged proboscis*—*big noses are funny across cultures and species!), the sociality of the animals, and the framing of bare earth, grass, and sky. But the underlying conservation story is also poignant. Saiga antelope populations have been decimated by hunting for their meat and horns*—*valued in Chinese medicine*—*dropping from a population of over one million in 1970s to approximately 50,000 today. Conservation efforts have stopped much of the hunting, but illegal poaching and highly*-*skewed sex ratios driven by culling the males for horns continue to threaten the species. The birthing season is a hopeful time for any conservation program, and it’s easy to project hope onto the animals seen here.”*

**Fig. 3:**
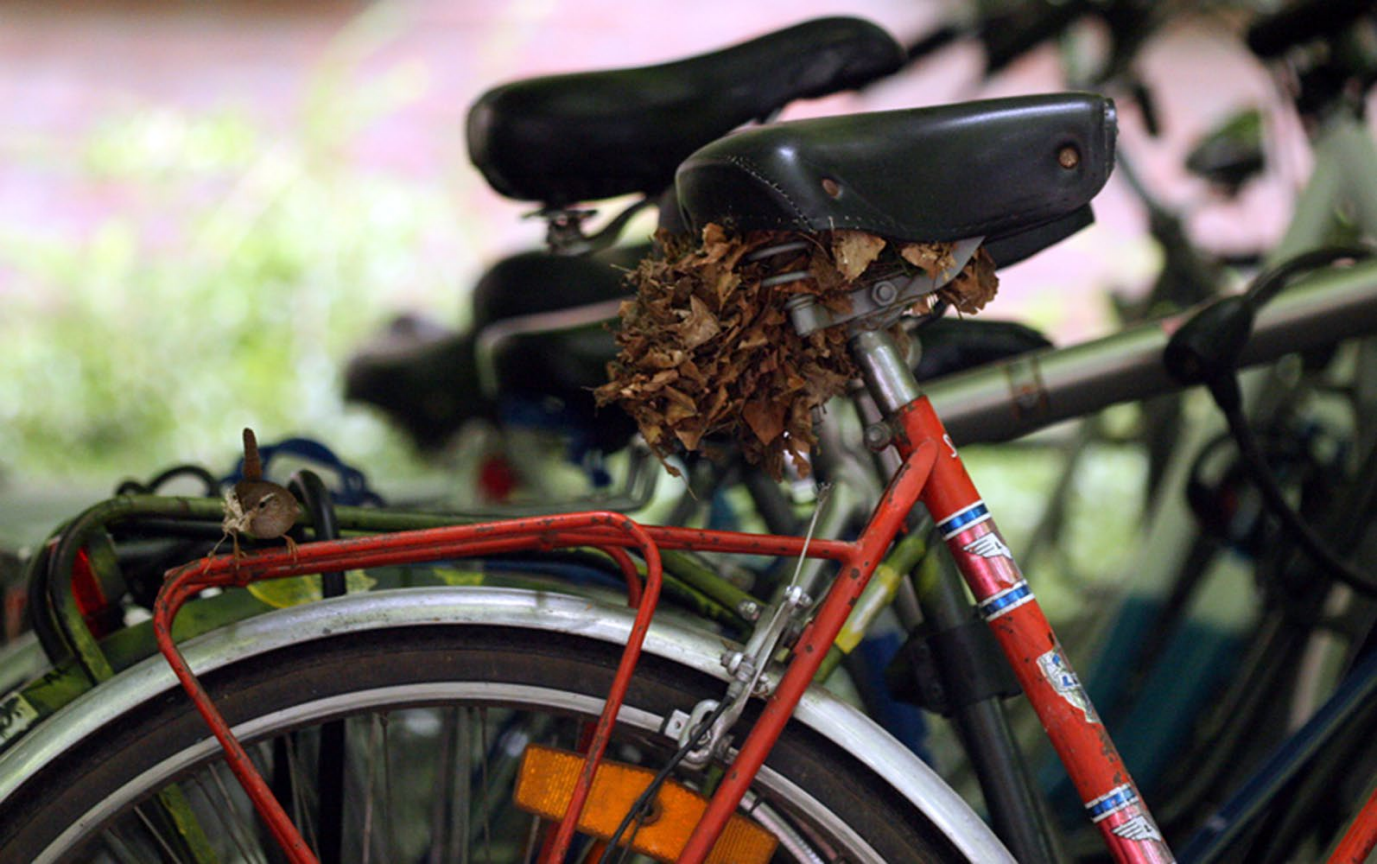
Second runner up: *“The Eurasian wren* (Troglodytes troglodytes)*, or simply ‘wren’, is a small brownish bird that is easily recognized by its stump tail, beautiful song and quite assertive territorial behavior. The male wren builds nests in bushes, tree trunks or any suitable hole, even in buildings. This male wren, adapted to busy campus life, decided to build a nest under the saddle of a parked bicycle. The wren initially started to line the nest with soft material (pictured), and this is usually an indication that a female has chosen the nest for breeding. Despite efforts to avoid disturbance by biology students and staff using the parking lot, the nest remained unoccupied and finally was blown out during a spring storm.”* Attribution: Raf Aerts

While we may often think of ecology and nature as being separate from human spaces, the image from second runner-up Raf Aerts (University of Leuven, Belgium) reminds us that sometimes the two can converge. In the photo he titled “*Troglodytes bicycletes*,” a Eurasian wren (*Troglodytes troglodytes*) has adopted a bike seat to build its nest (Fig. 3). This provides an example of urban ecology—a research area which our guest judge is dedicated to—and urges the viewer to reflect on the relationship and impact of human activity on the natural world.

## Behavioral and physiological ecology

The “Behavioral and Physiological Ecology” section received the highest number of submissions this year. The entries featured animal and plant species from all over the world and included a wide array of insects, birds, as well as large mammals like zebra and rhinoceros; we even saw a few representations of marine animals.

**Fig. 4:**
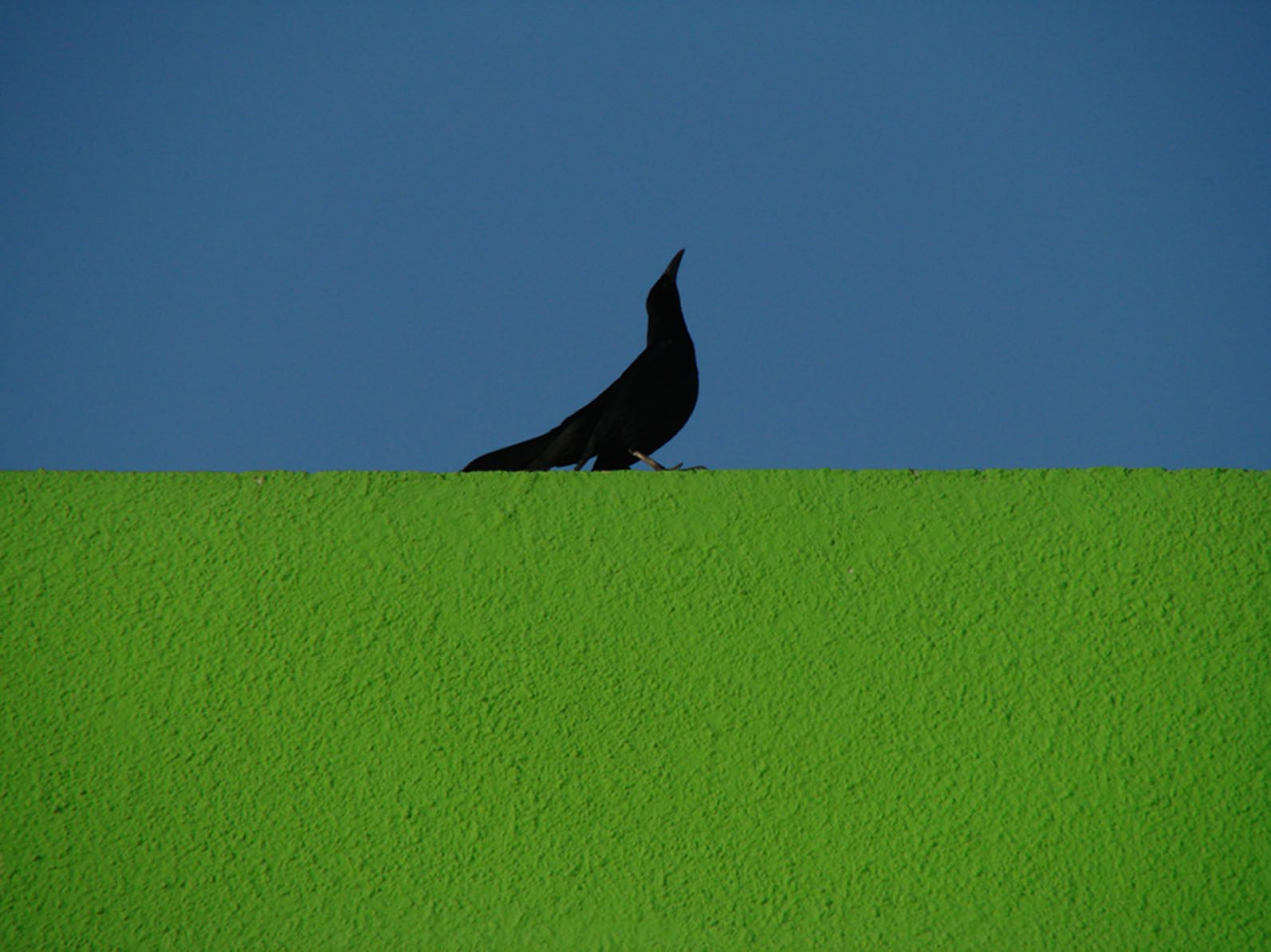
Winner, Behavioral and Physiological Ecology*: “In March 2013 I was in Chihuahua, Mexico, for a holiday trip. I took this picture in the center of the town, where some Great*-*tailed grackles were calling and displaying on a roof. This individual (likely an immature male according to its dark eye) was competing with other males without paying attention to people flowing in the crowded street below. The evening light made its dark silhouette stand out against the blue sky and the green wall.”* Attribution: Marco Zenatello

Section Editors Dominique Mazzi and David Hughes were both taken with the image by Marco Zenatello (Italian National Institute for Environmental Protection and Research), of a Great-tailed grackle he spotted on a city street (Fig. 4). As Dr. Mazzi points out, both the colors and the setting caught their interest: *“We selected the image from an excellent group of contenders showing the breadth and diversity of animal behavior. The displaying Great*-*tailed grackle captures many interesting aspects of behavior: a young male displaying in a cityscape, enthralled with himself and unaware of what is going on around him. The symmetry and balance of a few colors and clear lines, and the lack of distracting details make this visually stunning image grab the viewer’s attention.”*

## Conservation ecology and biodiversity research

**Fig. 5:**
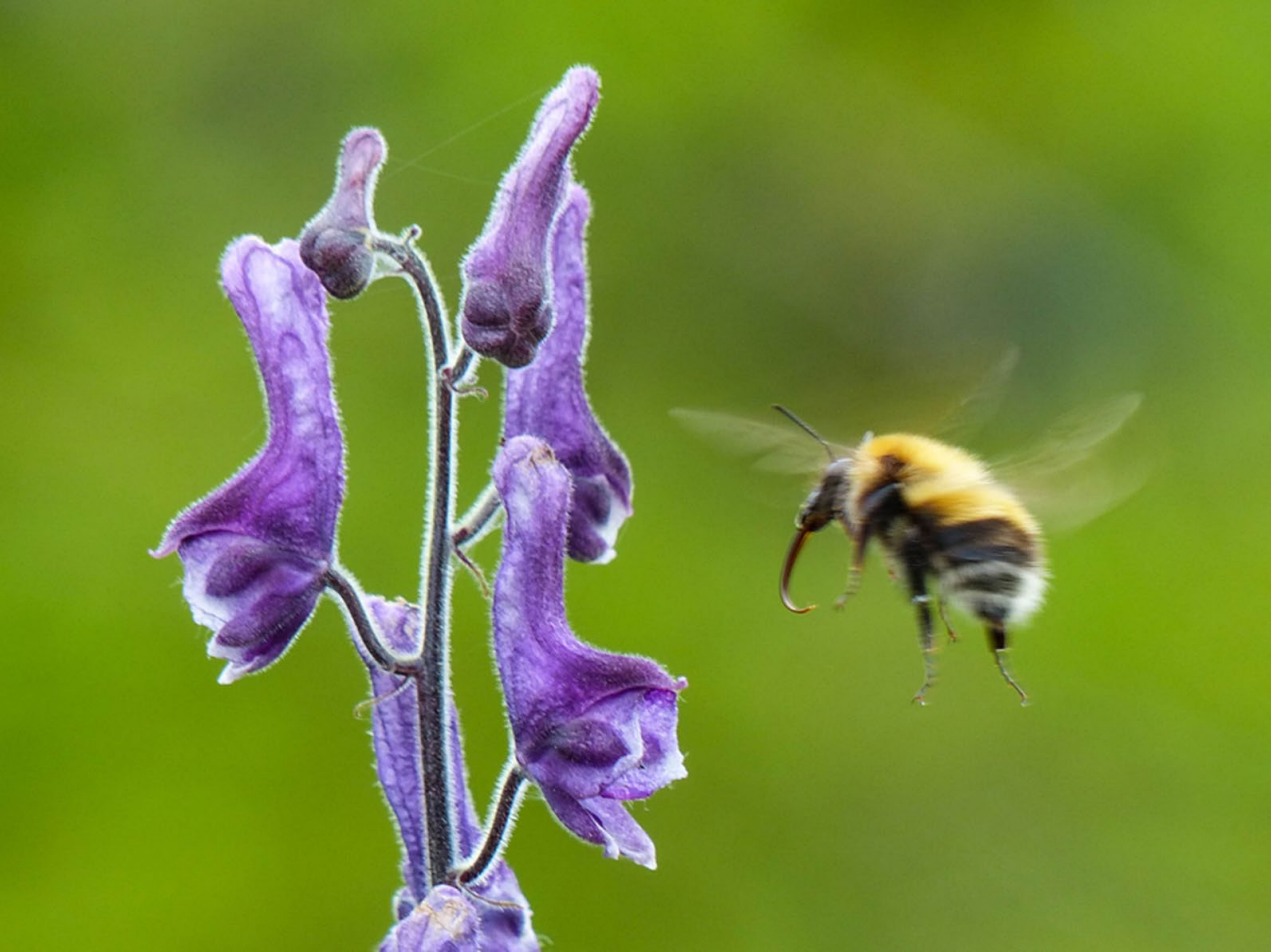
Winner, Conservation Ecology and Biodiversity Research: *“The intricate relationship between the long*-*tongued bumblebee* Bombus consobrinus *and the perennial herb* Aconitum lycoctonum *is a wonderful example of co*-*evolution.* B. consobrinus *is specially adapted to feed on* A. lycoctonum’s *long*-*spurred flowers and only exist within its range.* A. lycoctonum*, likewise, depends on the visits of* B. consobrinus *for pollination. There are cheaters in this system, however.* A. lycoctonum *is frequently subjected to nectar robbery by short*-*tongued bumblebees. They cannot reach the nectar the usual way, so instead they bite a hole on top of the flowers with their jaws. These holes produced by nectar robbing bumblebees can be seen on the flowers in the bottom part of the photo.”* Attribution, Elin Videvall

Co-section editors Luke Jacobus and Josef Settele chose a Conservation Ecology and Biodiversity Research winner that truly symbolizes the goals of the section. The snapshot by Elin Videvall (Lund University, Sweden) of the long-tongued bumble bee in pollinating action brings to mind progress in conservation efforts for declining biodiversity in bumble bee populations (Fig. 5). As key pollinators for many plant species, the conservation status of bees is critical not only for their species, but also for the plant species that rely on their pollinating activity.

Section Editor Josef Settele thought Elin’s photo was a great visual reminder of the continued efforts for bee conservation: *“The bumble bee picture was selected as it nicely represents the context of the very first assessment which was done within IPBES (the Intergovernmental Science*-*Policy Platform on Biodiversity and Ecosystem Services) [*4*], which dealt with pollination. This assessment was a major achievement on our road to bring biodiversity issue[s] much higher on the political agenda and rais[e] awareness at the UN level. More details of the report can be found here:*http://www.ipbes.net/sites/default/files/downloads/Pollination_Summary%20for%20policymakers_EN_.pdf*.”*

## Landscape ecology and ecosystems

**Fig. 6:**
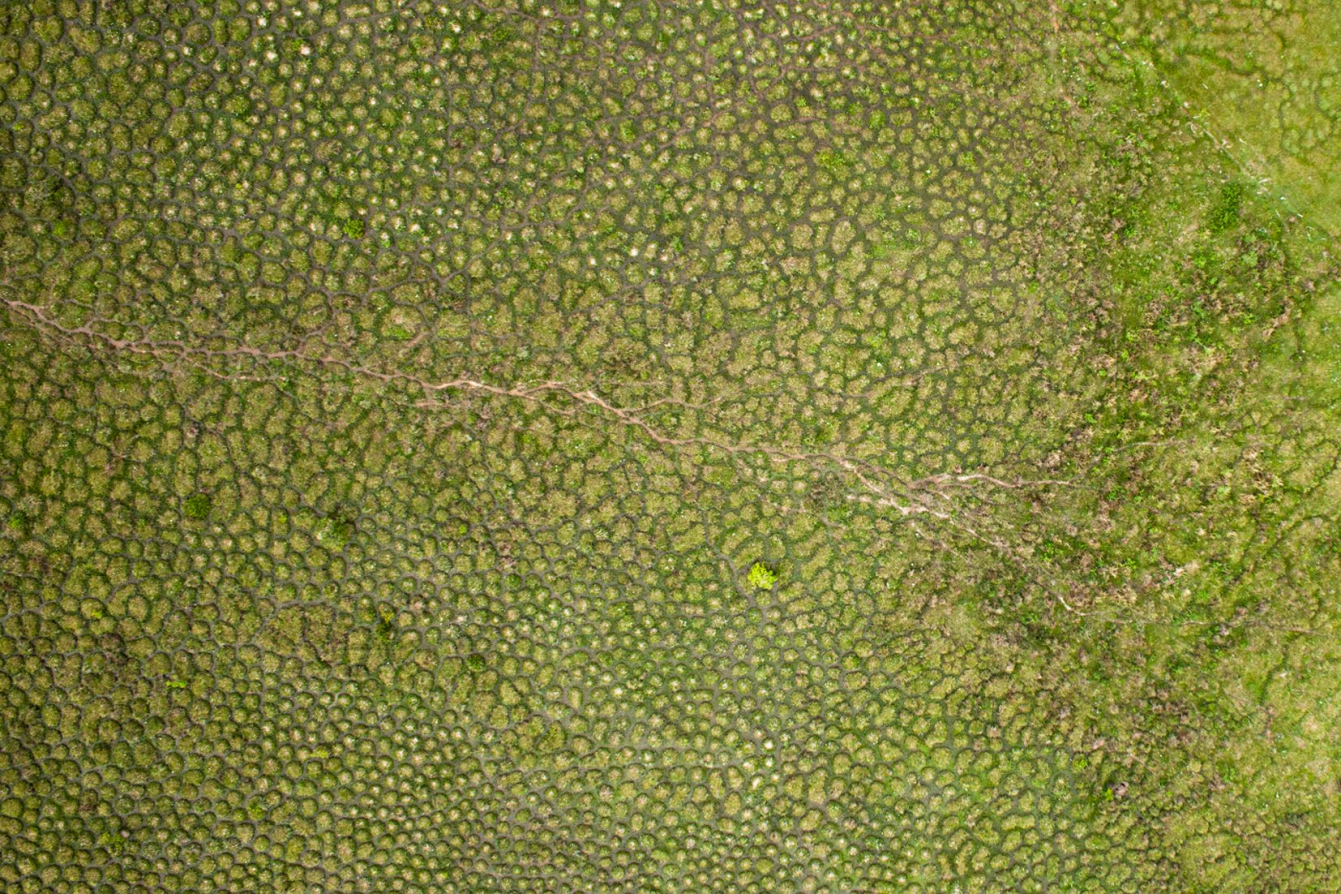
Winner, landscape ecology and ecosystems. Attribution: Delphine Renard, taken by PIXY™ drone

Our pick for the winner of the “Landscape Ecology and Ecosystems” section exploited drone technology to generate this striking aerial view of *surales* structures in South America (Fig. 6). Not only are these geometric land structures visually interesting, they arise through a fascinating ecological process—the foraging activity of earthworms.

Delphine Renard, of the University of California Santa Barbara, who submitted the photo, describes her research group’s discovery of the unusual origins of these *surales*: *“Thishigh*-*resolution aerial photographwas taken inColombia using a remote*-*controlled drone (picture credit: Delphine Renard, PIXY™ drone). It shows hundreds of densely*-*packed, regularly spaced earth mounds. The round mounds in this photograph are about 1.5*–*2 meters in diameter and 0.50 meters in height. Although descriptions of these mounds, called “*surales*”, date back to the 1940s, their ecology was virtually unknown.Using my own aerial photographs*—*including the one here*—*and satellite imagery from Google Earth, I surveyed* surales *landscapes across the savanna grasslands of the Orinoco Llanos in Colombia and Venezuela.* Surales *are much more common that we at first thought. Indeed, they are present in an area covering about 75,000* *km*^*2*^*, e.g. larger than the area of the Republic of Ireland.Combining data on soil physical and chemical properties, soil macrofauna and vegetation, we showed the key role played by a still*-*undescribed species of earthworm (*Andiorrhinus sp*.)* *in the formation of* surales. *This picture reveals how stunning the patterns created by accumulated earthworm poop can be, seen from the air. The story of *surales *was recently published* [5].”

Matthew Palmer noticed the methodological significance of this image, and reminds us that along with the *“beautiful landscape pattern, [it] shows the benefits of drone technology for ecology, in this case leading to a potential explanation for a formerly mysterious pattern.”*

## Community, population, and macroecology

**Fig. 7:**
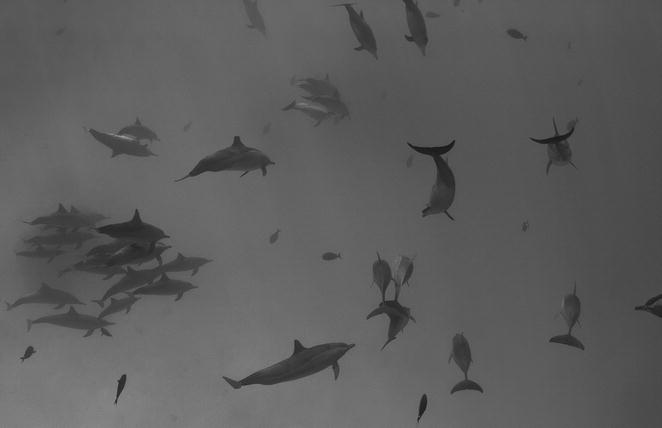
Winner, Community, Population, and Macroecology: *“I was snorkeling in a remote lagoon in the Sudanese Red Sea when I was suddenly surrounded by hundreds of spinner dolphins. The school stayed around for hours, visibly enjoying the interaction with snorkelers in the water. The school was clearly subdivided into dozens of smaller groups of either females with their offspring or adult males.”* Attribution: Julia Spät

The winner of the “Community, Population and Macroecology” section brought us a glimpse into an animal community that we land-dwellers don’t usually see. Julia Spät (University of Cambridge, UK) presents this dramatic black and white image of a school of dolphins that she captured while snorkeling (Fig. 7).

Section Editor Simon Blanchet recognizes this as one of the perks of life as an ecologist: *“it shows the chance we have to do that job and the proximity we can have sometimes [to] the wildlife. This is in my opinion a motivating image for young scientists and for more experienced scientists, since sometimes it is important to come back to the roots and remind ourselves why we are doing this amazing job.”*

## Editor’s pick

**Fig. 8:**
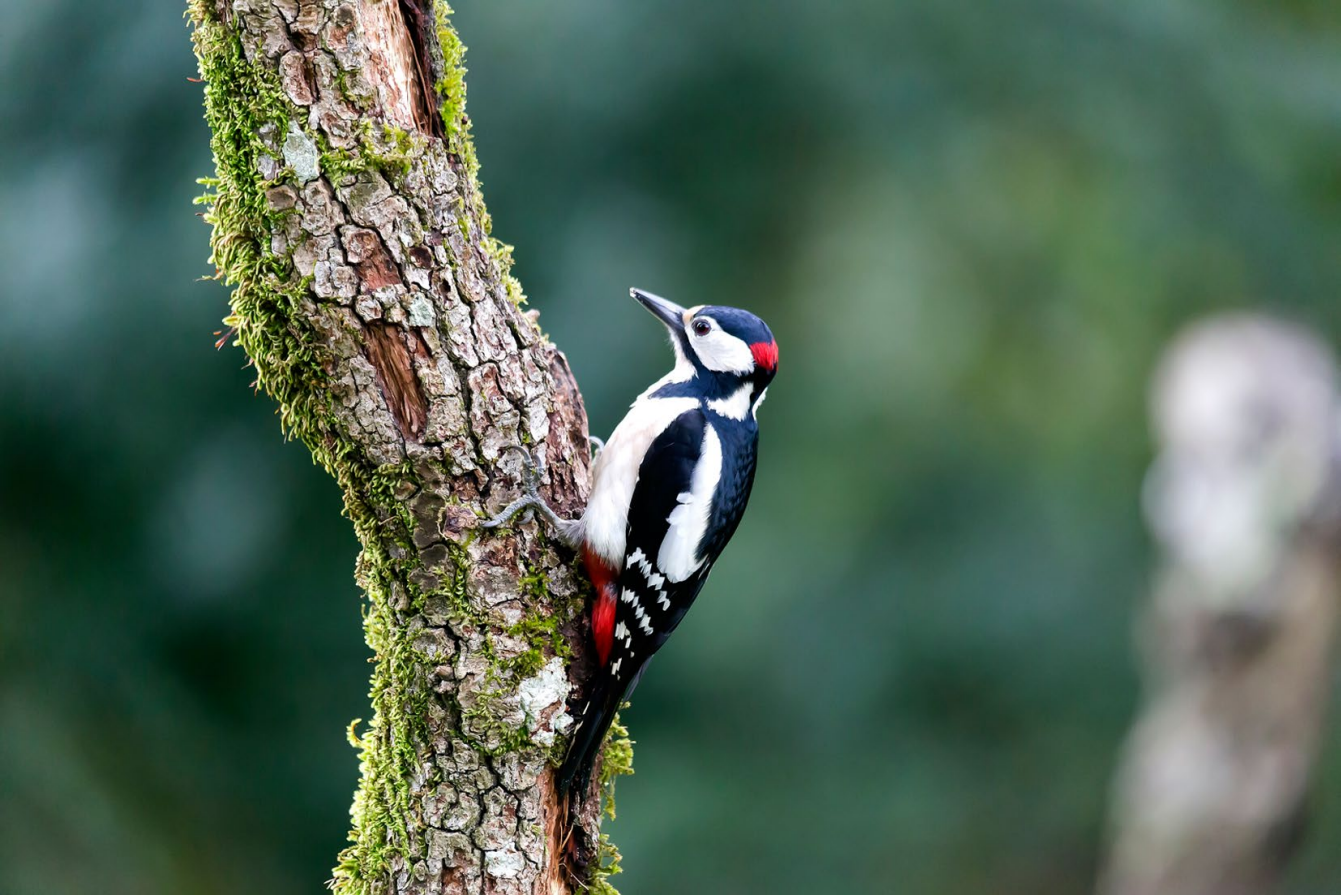
Winner, Editor’s Pick.*“Tree trunk of Quercus faginea* Lam. subsp. broteroi (Cout.) A. Camus *with the bryophytes* Homalothecium sericeum (Hedw.) Schimp, Nogopterium gracile (Hedw.) Crosby & W.R. Buck *and the great spotted woodpecker* [(Dendrocopos major L., 1758)]. *Location: Ourém. Portugal. 9*-*3*-*2016.”* Attribution: César Garcia

Nature produces fascinating patterns—like the precise geometry of the great spotted woodpecker’s feathers. The sharp pattern of the bird alone was enough for the image by César Garcia (University of Lisbon, Portugal) to catch our attention (Fig. 8); however, as is often the case in ecology, this prominent feature is hardly the full story. The star subject is actually the tree trunk, which is supporting concurrent ecological interactions—with the visiting bird, as well as with the resident moss. Furthermore, this image hints at an unseen community: the woodpecker will presumably use the tree for its signature “peck,” to announce its presence to other woodpeckers in the area.

## Highly commended

### Vibrant colors

Many images were immediately arresting because of their incredible and often unexpected color schemes. The range of colors found in nature are illustrated at the ecosystem level, shown the underwater seascape by Benjamin Geffroy (Additional file 1), and at the organismal level of two different colorful lorikeet species by Abd Al-Bar Al-Farha (Additional file 2).

In another image (Additional file 3), the vibrant blue of the iceberg enforces its presence in the background of a Chinstrap penguin, reminding us of the perseverance required for these animals to thrive in harsh conditions. With similar color palettes but different climates, a killer whale emerges from bright blue Australian waters (Additional file 4), and silhouettes of shearwaters above the sea break through a glowing Mediterranean horizon (Additional file 5).

In some cases, colors may initially catch a viewer’s attention, but on further inspection are secondary to the bigger story behind the image. The gradient hues in the frailejón succulent drew in guest judge Matthew Palmer, but he also noted the structural features that represent its need to survive adverse conditions in its changing environment (Additional file 6).

The significant variation in maize lends itself to creativity: Somnath Roy and colleagues produced a beautiful landscape using colorful diverse maize landraces (Additional file 7)—demonstrating a whimsical and unusual application of ecological research.

## Ecology and technology

A theme of this year’s competition was the interaction of nature with human activity and manmade technology, which produced some unique images.

Some very enjoyable moments were captured when the animal subjects seem to acknowledge or even pose for the camera—such as the enthusiastic proboscis monkey seen by David Constantini (Additional file 8), or the herd of waterbuck that give a skeptical gaze at the motion-capturing camera set up in the Gorongosa National Park (Additional file 9). Matthew Palmer described this scene as “*a wonderful serendipitous moment*—*captured without an actual photographer but with lovely composition. This image also illustrates both citizen science and the recovery of wildlife following armed conflict.”*

Similarly, Lawrence Reeves used an ultraviolet light to attract a swarm of moths, creating a remarkably composed image that not only recreates the behavior of moths in nature but also demonstrates a practical tool used by field researchers, to keep the moths away from their equipment (Additional file 10). In another unusual application of technology in ecological research, Jean-Luc Jung and colleagues visualized the “acoustic fat” of a porpoise head using Magnetic Resonance Imaging (MRI), which generated an eerie image resembling a human face (Additional file 11).

While the wren in Fig. 3 is an example of nature “reclaiming” human-made spaces, in some cases human spaces are intentionally built around nature—such as the bridge dwarfed by its surrounding bamboo plants at the National Coffee Park in the rural area of Montenegro, Quindío, Colombia, in the submission from Arnubio Valencia Jimenez (Additional file 12).

Finally, another recurring theme in this year’s winning images was the notable human efforts toward ecological conservation. In Bethany Clark’s image (Additional file 13), a tracking tag on a gannet’s leg aids studies of the behavior and travel patterns of individuals and populations, in order to better evaluate management options.

## Unique interactions

Many of the submissions portrayed specialized relationships between species that have likely evolved and developed over long periods of time. One of this year’s outstanding examples is Ethan Newman’s shot of the proboscid fly approaching the *Nerine humilis* plant—the unique anatomy of both the fly and the flower have adapted to optimize pollination efforts (Additional file 14).

A more subtle relationship exists in the fungal attachment to the *Scilla bifolia* flower, giving a dusty charcoal coating to the otherwise lavender petals of infected flowers. This relationship is intriguing as it not only alters the aesthetic appearance of the petals, but also affects the biology, leading to sterilization of the host [6–8] (Additional file 15). Another surprising parasitic relationship is seen in the lurking eyes of parasitic *Heteropelma amictum* wasp pupa inside the host cuticle of a *Callimorpha dominula* caterpillar pupa (Additional file 16).

Section Editor David Hughes, an ant expert, admired a photo capturing myrmecophily—or mutual association with ants—by Arpan Kumar Parui (Additional file 17). In this photo, weaver ants interact with homoptera nymphs on a plant.

The characteristic sandy imprint on the Indo-Pacific coast photographed by Ulisse Cardini (Additional file 18) is the result of foraging and burrowing behavior by sand bubbler crabs—illustrating interplay between predator, prey, and environment in a single ecosystem.

## Conclusion

We were amazed once again by the variation and talent in the submissions to the 2016 Image competition, and are thrilled to use this event to celebrate ecology and the research activities of ecologists worldwide. We hope that our readers enjoy this collection of images, and that they serve as inspiration for closer observation and reflection of the animals, plants, and geological phenomena with which we share our natural surroundings.

## Additional files

10.1186/s12898-016-0090-z “This photograph shows fish (mostly *Characidae* of the genus *Astyanax* and *Odontostilbe*) in a clear tributary of the Cuiabá River (Nobres, Mato Grosso, Brazil) famous for ecotourism. As part of my research, I was investigating gene expression, behavior and hormones in those fish following high tourism exposure during the soccer World Cup in 2014.” Attribution: Benjamin Geffroy (Federal University of Mato Grosso, Brazil).
10.1186/s12898-016-0090-z “This image was taken in Adelaide Botanic garden [in 2016]. Rainbow lorikeets are such colorful parrots that it is hard to mistake them for other species. The related Scaly-breasted lorikeet is similar in size and shape, but can be distinguished by its all-green head and body.” Attribution: Abd Al-Bar Al-Farha (University of Adelaide, Australia).
10.1186/s12898-016-0090-z “Seconds before the shot, this Chinstrap penguin (*Pygoscelis antarctica*) came out of the water after one of its many daily foraging trips that are performed as part after their chicks are born. There is not a second to lose, so after drying its wings a little bit, he would walk up the hill, switch positions with his couple and deliver the most precious goods in this harsh environment. Once the mother is back he will go back to the water and do the best he can one more time. Energy acquisition and a successful foraging behavior is all that matter at this point of the breeding season.” Attribution: Renato Borrás-Chávez (Pontifical Catholic University of Chile).
10.1186/s12898-016-0090-z “This was taken during my most recent field season studying the killer whales (*Orcinus orca*) at Bremer Canyon, Western [Australia] for my PhD. We were with a group of approximately 7 individuals taking fin shots, when all of a sudden the group had a change of direction and picked up their speed. They were on the hunt! We observed them charging and porpoising through the water at high speed chasing down their next meal. What ensued was a successful predation on another cetacean. Seeing them in all their might and glory and speed, you certainly gain respect for this King of the Ocean.” Attribution: Rebecca Wellard (Curtin University, Western Australia).
10.1186/s12898-016-0090-z “Linosa Island in Italy holds the second largest colony of Scopoli’s shearwaters (*Calonectris diomedea*) in the Mediterranean. Every day, before the sunset, shearwaters gather in large groups a few (kilometers) off shore, rafting on the sea patiently waiting for night when they can fly ashore.” Attribution: David Costantini (University of Antwerp, Belgium).
10.1186/s12898-016-0090-z “This picture was taken in Colombia, at the “Cruz Verde” Páramo in February 2015. The species shown here (*Espeletia* sp.) commonly called “frailejón” is an important and common element of the Páramo ecosystem. Their succulent leaves and thick hair layer are adaptations to cold and extreme temperatures characteristic of Páramos. Currently, the genus *Espeletia* comprises several species, some of them are highly threatened by climate change and human activities such as agriculture.” Attribution: Francisco Javier Velásquez Puentes (University of Gothenburg, Sweden).
10.1186/s12898-016-0090-z “Maize (*Zea mays*) is the second most important crop of northeastern (NE) region of India. It [may] be considered as a secondary center of origin after Mexico. Occurrence of Sikkim primitive -1, a primitive and highly prolific maize landrace, in this region supports this theory. Many studies have shown that [diversity] of maize landraces in NE India is huge. However, a comparison with the global collections is required. [NE] India is dominated by (around 97 % of the total population) tribal communities. The soil and environmental heterogeneity of this region is also huge. Altogether, the ethnolinguistics and environment of NE India [led] to development [of] plant diversity. Around 5000 germplasm accessions of maize [have] already been collected and conserved from eight states of NE India. All eight states have maize resources, however the diversity is considerably high in Sikkim, Arunachal Pradesh, Nagaland, Manipur and Mizoram. Among the many, notable variation [has been] observed for [traits] such as cob size and shape, color of the kernels, plant architecture, amylose and protein content in the kernels, prolificacy, and more. The indigenous farms still rely on the traditional composite landraces for their food security. This photo represents the diversity in kernel morphology of some maize landraces conserved in ex situ gene bank of ICAR-National Bureau of Plant Genetic Resources, Regional Station, Umiam, Meghalaya, India.” Attribution: Somnath Roy (Indian Council of Agricultural Research, India).
10.1186/s12898-016-0090-z “Dominant males of the proboscis monkey (*Nasalis larvatus*) found only on the island of Borneo defend strenuously their females against intruders.” Attribution: David Costantini (University of Antwerp, Belgium).
10.1186/s12898-016-0090-z “This photo of a herd of waterbuck in the morning mist was taken by a motion-detecting trail camera in the Gorongosa National Park in Mozambique. A network of fifty trail cameras were set up by Paola Bouley, a researcher who is studying how the lion population is rebounding in Gorongosa after decades of war devastated wildlife populations. Hundreds of thousands of photos that she and her team have collected are available for citizen scientists to help her identify on the website WildCam Gorongosa. Waterbuck are a common sight in Gorongosa as their population has exploded to over 34,000 individuals up from only a few hundred after the war. Scientists are studying the waterbuck population to learn why they are experiencing such rapid growth.” Attribution: Chuck Schultz (Science Education Department, Howard Hughes Medical Institute).
10.1186/s12898-016-0090-z “Every year, the monsoon rains of late summer bring the Sonoran Desert to life. Most of the region’s insect species time the emergence of adult life stages with the arrival of the monsoons. Entomologists often use lights as lures for insects. Light trapping in southeastern Arizona during this time of year attracts insect numbers on par with the tropics. A typical light trap consists of a white bed sheet hung perpendicular to the ground, with a bright light in front of it. At the end of the night, when the lights go off, we used a dim UV light to attract the swarms of moths and other insects so they don’t dive bomb our head lamps, or fill the open doors of our vehicles. Using a dim light to attract the moths from the sheet keeps them out of trouble and distracted, so that the equipment can be packed. This photo was taken at the end of a night of collecting, as the moths swarmed the UV light.” Attribution: Lawrence Reeves (University of Florida).
10.1186/s12898-016-0090-z “This picture was taken in the frame of a pluridisciplinary study of the biochemical and anatomical basis of echolocation functioning in toothed whales. As a part of this study, specialists of medical imaging of the Brest Hospital (France) use MRI (Magnetic Resonance Imaging) to decipher the complex anatomical structure of “acoustic fat” tissues (melon and mandibular fats) in odontocetes. It [was] during such a working session (grouping Marion Arribart, veterinary student at ONIRIS, Nantes; Douraied Ben Salem and Julien Ognard, medical doctors, specialists of medical imaging, CHRU of Brest; and Jean-Luc Jung, molecular biologist and geneticist, BioGeMME laboratory, University of Brest), that Douraied Ben Salem had his attention drawn by this strange and surprising image of a harbor porpoise head, appearing on the screen, among other more classical pictures. The MRI captured the harbor porpoise head as an amazing kind of tribal mask, where the melon and the mandibular fats are visible, inter-bones spaces and the pharynx appear as eyes and mouth, and mandibulae look like very visible earrings, thus giving a strange artistic dimension to this scientific picture. The picture has not been modified or altered in any way.” Attribution: Jean-Luc Jung (University of Brest, CHRU of Brest, ONIRIS).
10.1186/s12898-016-0090-z “Bamboo plants in perfect harmony with buildings of interest to human communities. Prevailing respect for nature and its species.” Attribution: Arnubio Valencia Jimenez (University of Caldas, Colombia).
10.1186/s12898-016-0090-z “G407 is an immature gannet that was fitted with a unique colored band and a GPS logger, which is just visible on the tail. Steve Votier and I tagged this bird as a part of project run by University of Glasgow’s Jana Jeglinski, which investigates the movements of gannets before they settle down to breed, as very little is known about this 4–5 year period. These solar-powered loggers ‘text’ GPS data via the mobile phone network, so the bird can be tracked in real time until the bird naturally shed the tag as she replaced her tail feathers. After leaving RSPB Grassholm Island in West Wales, a colony of 36,000 breeding pairs, she travelled to Ireland, Cornwall and France!” Attribution: Bethany Clark (University of Exeter, UK).
10.1186/s12898-016-0090-z “The long proboscid fly, *Prosoeca longipennis* approaches the inflorescences of *Nerine humilis* (*Amaryllidaceae).* The distribution of the flies is confined to just a few *N. humilis* populations in South Africa, and the unusually long styles and anthers of those *N. humilis* populations are locally adapted to receive and deposit pollen on the abdomens of the flies.” Attribution: Ethan Newman (University of Stellenbosch, South Africa).
10.1186/s12898-016-0090-z “Beauty is in the eye of the beholder: *Antherospora scillae* on *Scilla bifolia* in an early spring beech grove. Most people rejoice in the beauty of early bloomers like squills or snowdrops heralding spring. For parasitologists delight is even bigger if plants are infected by organisms that live at the expense of their host. The *Antherospora* smut fungi sporulate in the anthers and on the surface of the inner floral organs of different *Hyacinthaceae* replacing the pollen and thus sterilizing the host plant. Quite recently they were shown to represent a distinct phylogenetic lineage that now includes 12 species some of which are morphologically similar but phylogenetically different and strictly host species specific. The mind of non-parasitologists may be put at ease: Hudson et al. (2006) considered a healthy system to be one that is rich in parasite species!” Attribution: Matthias Lutz (University of Tübingen, Germany).
10.1186/s12898-016-0090-z “Metamorphosis of a parasitoid in its host. Typically, it is the developing pair of compound eyes that, due to their dark pigmentation, are first distinguishable in a moth pupa during its metamorphosis. Sometimes, however, these eyes do not belong to the moth but to a parasitoid wasp. In the image presented, it is the pupa of *Heteropelma amictum* (Hymenoptera: Ichneumonidae), that is developing in its host, *Callimorpha dominula* (Lepidoptera: Erebidae). Attribution: Franziska Bauer (Senckenberg Natural History Collections Dresden, Germany).
10.1186/s12898-016-0090-z “Myrmecophily is a well-known mutualistic interaction between ants and homopterans. Weaver ants (*Oecophylla smaragdina*) are behaviorally very dominant species of ants, and they generally predate upon other insects. During my 3 years [of] field work in a mixed deciduous forest in India, I found *Oecophylla* ants to mostly have protein diet & using baiting experiment[s], I found them to prefer protein baits over honey baits. Also I didn’t find any *Oecophylla* individual tending aphids in the forest. In my garden, however, I found *Oecophylla* ants to tend homoptera nymphs (depicted here) on a regular basis. Perhaps the availability of protein foods is an important regulating factor of their homoptera rearing behavior. I also found the ants carrying the hopper nymphs with their mandibles and placing them at different parts of the plant.” Attribution: Arpan Kumar Parui (University of Calcutta, India).
10.1186/s12898-016-0090-z “Sand bubbler crabs live on soft shores of the tropical Indo-Pacific. They live in burrows in the sand, where they hide during high tide. During low tide, while searching the sand for food, they form characteristic sand pellets which cover the sand. They play an important ecological role as deposit feeders and bioturbators, and have been shown to affect the productivity of sandy shores. They move in large groups and scout the beach radially from their burrows, creating intricate and characteristic patterns of pellets as they proceed.” Attribution: Ulisse Cardini (University of Vienna, Austria).

## References

[1] Harold S, Wong Y, Baguette M, Bonsall MB, Clobert J, Royle NJ et al. BMC ecology image competition: the winning images. BMC Ecol. 2013;13:6. http://www.biomedcentral.com/1472-6785/13/6. 10.1186/1472-6785-13-6 PMC3606310 23517630

[2] Harold S, Henderson C, Baguette M, Bonsall MB, Hughes D, Settele J. BMC ecology image competition 2014: the winning images. BMC Ecol. 2014;14:24. http://www.biomedcentral.com/1472-6785/14/24. 10.1186/s12898-014-0024-6 PMC4236560 25178017

[3] Potenski C, Porzecanski AL, Baguette M, Clobert J, Hughes D, Settele J. BMC ecology image competition 2015: the winning images. BMC Ecol. 2015;15:22. http://www.bmcecol.biomedcentral.com/articles/10.1186/s12898-015-0053-9. 10.1186/s12898-015-0053-9 PMC4517399 26219534

[4] IPBES. Summary for policymakers of the assessment report of the Intergovernmental Science-Policy Platform on Biodiversity and Ecosystem Services on pollinators, pollination and food production. In: Potts SG, Imperatriz-Fonseca VL, Ngo HT, Biesmeijer JC, Breeze TD, Dicks LV, Garibaldi LA, Hill R, Settele J, Vanbergen AJ, Aizen MA, Cunningham SA, Eardley C, Freitas BM, Gallai N, Kevan PG, Kovács-Hostyánszki A, Kwapong PK, Li J, Li X, Martins DJ, Nates-Parra G, Pettis JS, Rader R, Viana BF, editors. 2016. p. 1–28.

[5] Zangerlé A, Renard D, Iriarte J, Suarez Jimenez LE, Adame Montoya KL, Juilleret J (2016). The surales, self-organized earth-mound landscapes made by earthworms in a seasonal tropical wetland. PLoS One.

[6] Bauer R, Lutz M, Begerow D, Piatek M, Vanky K, Bacigalova K, Oberwinkler F (2008). Anther smut fungi on monocots. Mycol Res.

[7] Piçtek M, Lutz M, Smith PA, Chater AO (2011). A new species of Antherospora supports the systematic placement of its host plant. IMA Fungus.

[8] Hudson PJ, Dobson AP, Lafferty KD (2006). Is a healthy ecosystem one that is rich in parasites?. Trends Ecol Evol.

[9] Creative Commons Attribution License (https://creativecommons.org/licenses/by/4.0/).

