# Supplementary figures and images for: BMC Ecology Image Competition 2016: the winning images
Source: BMC Ecol. 2016 Aug 9;16:34. doi: 10.1186/s12898-016-0090-z (PMC4977851; doi:10.1186/s12898-016-0090-z)

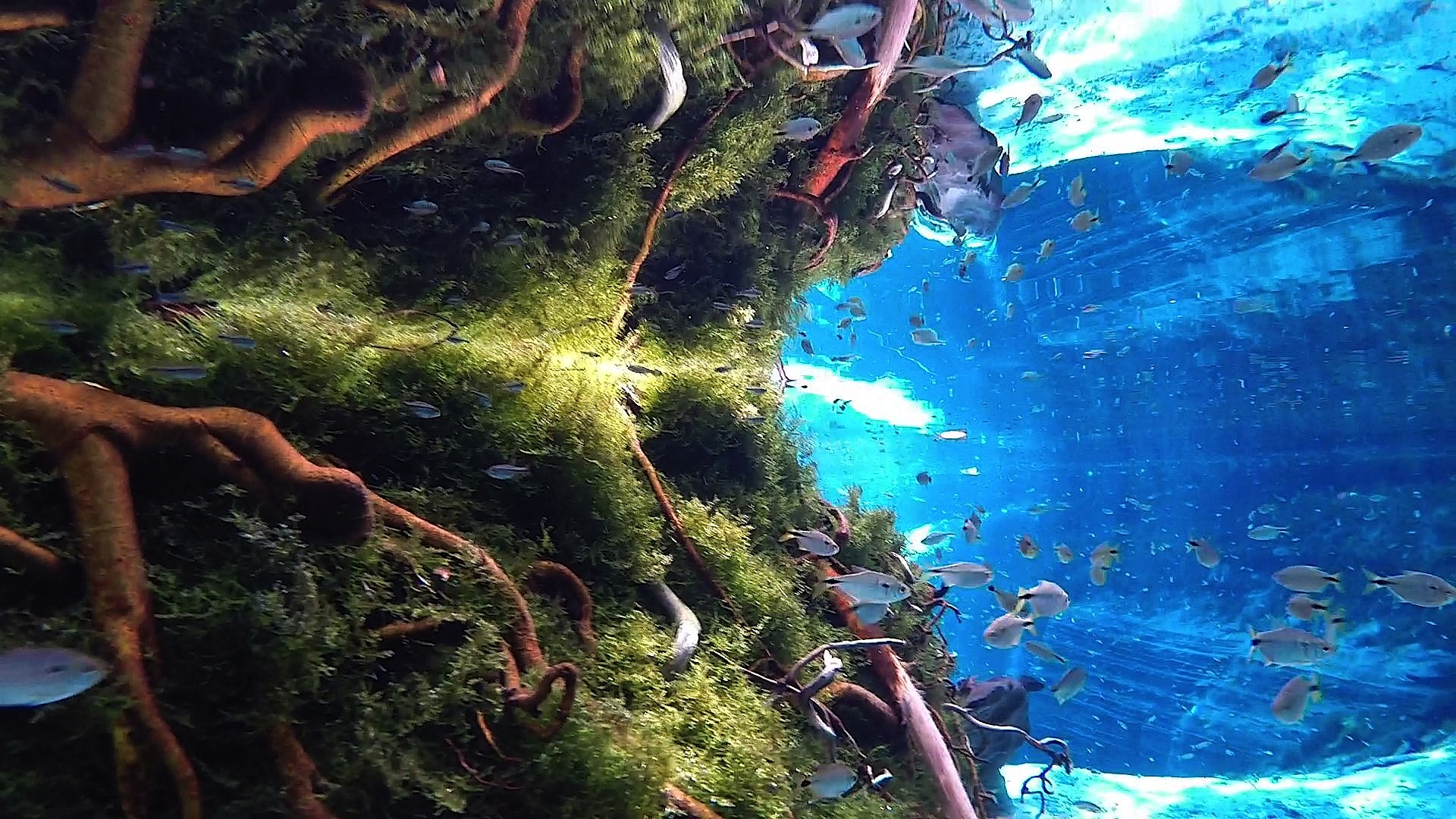

Supplement: Supplementary file 1 — 10.1186/s12898-016-0090-z “This photograph shows fish (mostly Characidae of the genus Astyanax and Odontostilbe) in a clear tributary of the Cuiabá River (Nobres, Mato Grosso, Brazil) famous for ecotourism. As part of my research, I was investigating gene expression, behavior and hormones in those fish following high tourism exposure during the soccer World Cup in 2014.” Attribution: Benjamin Geffroy (Federal University of Mato Grosso, Brazil). [file 12898_2016_90_MOESM1_ESM.jpg]

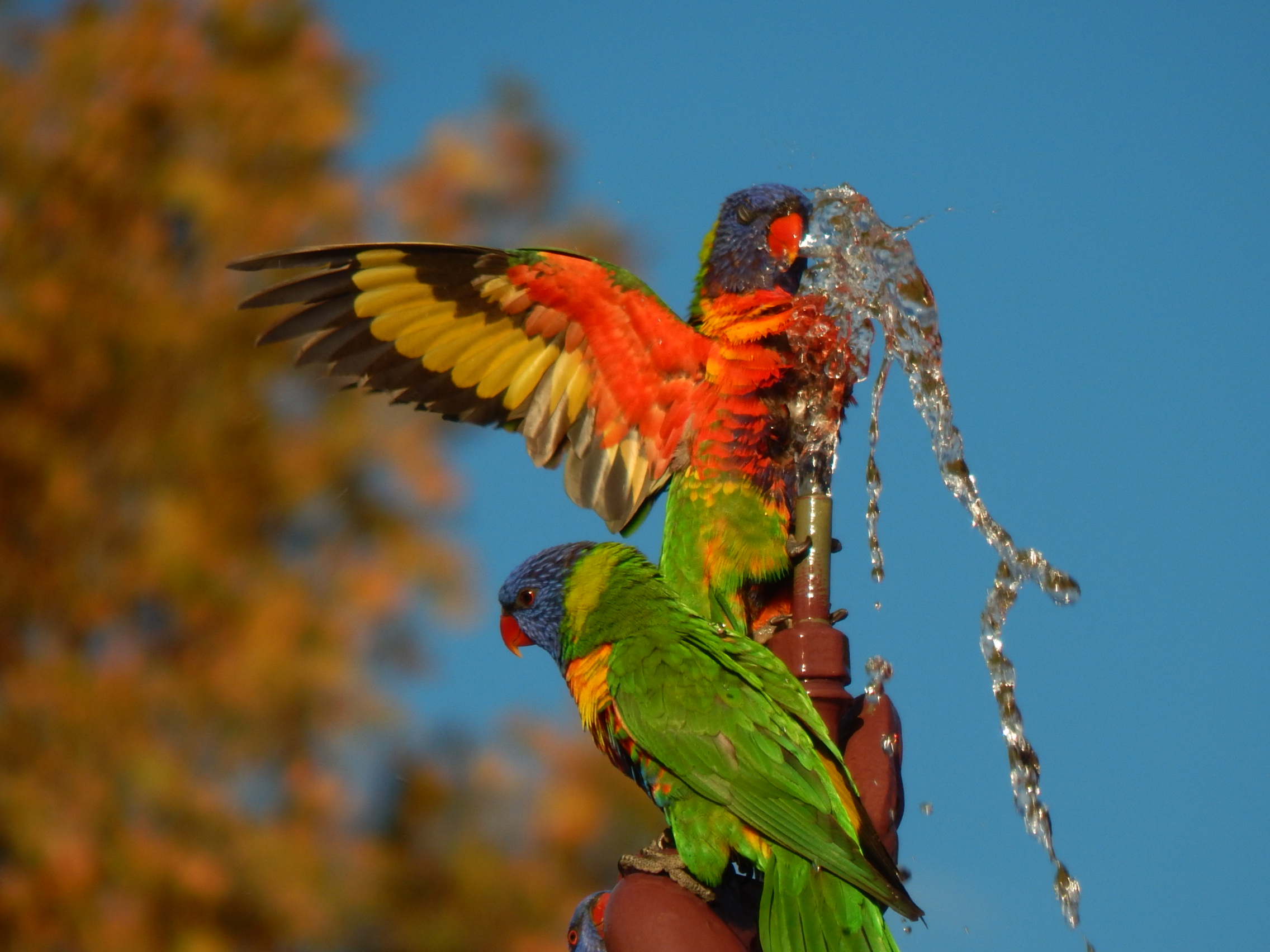

Supplement: Supplementary file 2 — 10.1186/s12898-016-0090-z “This image was taken in Adelaide Botanic garden [in 2016]. Rainbow lorikeets are such colorful parrots that it is hard to mistake them for other species. The related Scaly-breasted lorikeet is similar in size and shape, but can be distinguished by its all-green head and body.” Attribution: Abd Al-Bar Al-Farha (University of Adelaide, Australia). [file 12898_2016_90_MOESM2_ESM.jpg]

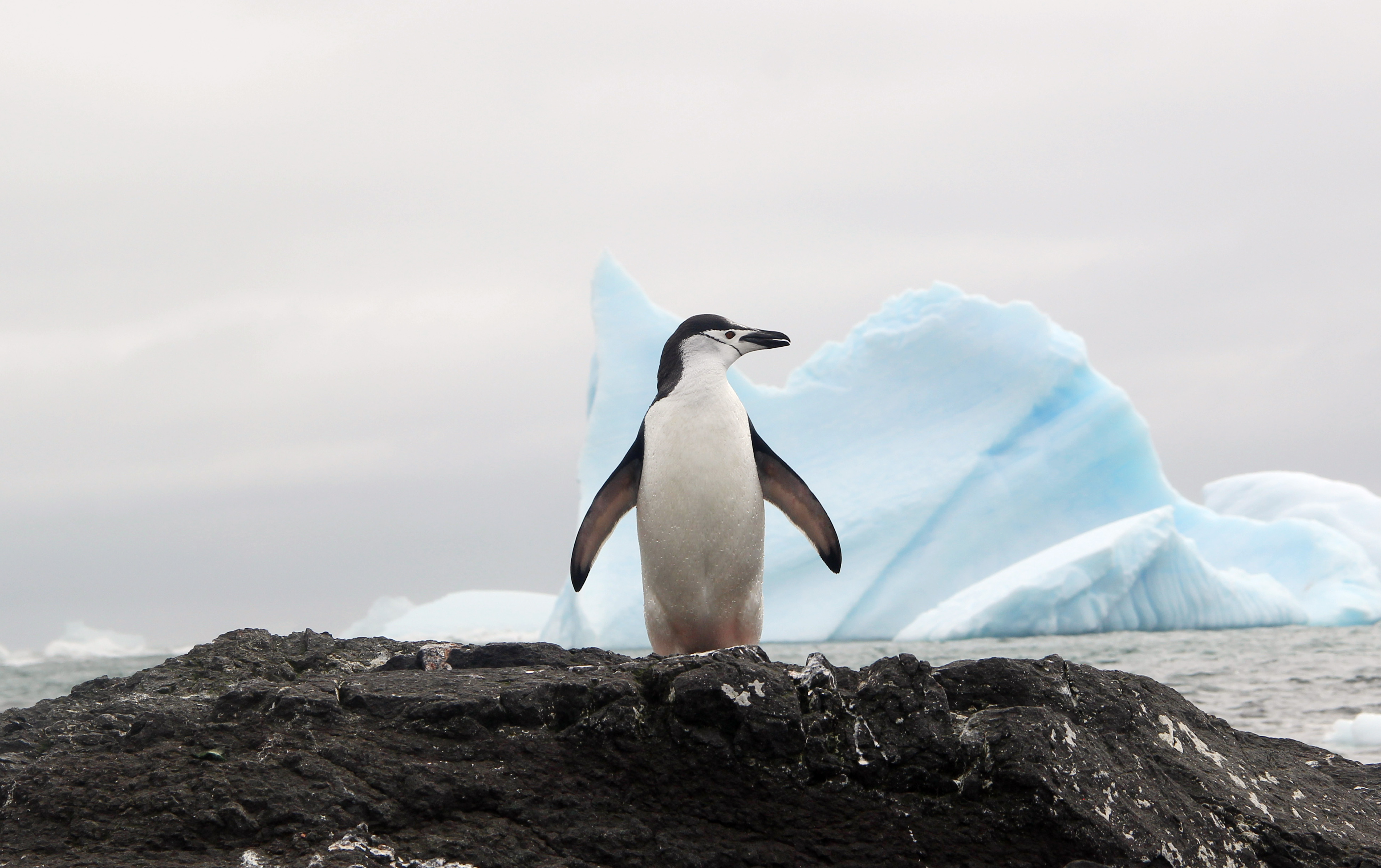

Supplement: Supplementary file 3 — 10.1186/s12898-016-0090-z “Seconds before the shot, this Chinstrap penguin (Pygoscelis antarctica) came out of the water after one of its many daily foraging trips that are performed as part after their chicks are born. There is not a second to lose, so after drying its wings a little bit, he would walk up the hill, switch positions with his couple and deliver the most precious goods in this harsh environment. Once the mother is back he will go back to the water and do the best he can one more time. Energy acquisition and a successful foraging behavior is all that matter at this point of the breeding season.” Attribution: Renato Borrás-Chávez (Pontifical Catholic University of Chile). [file 12898_2016_90_MOESM3_ESM.jpg]

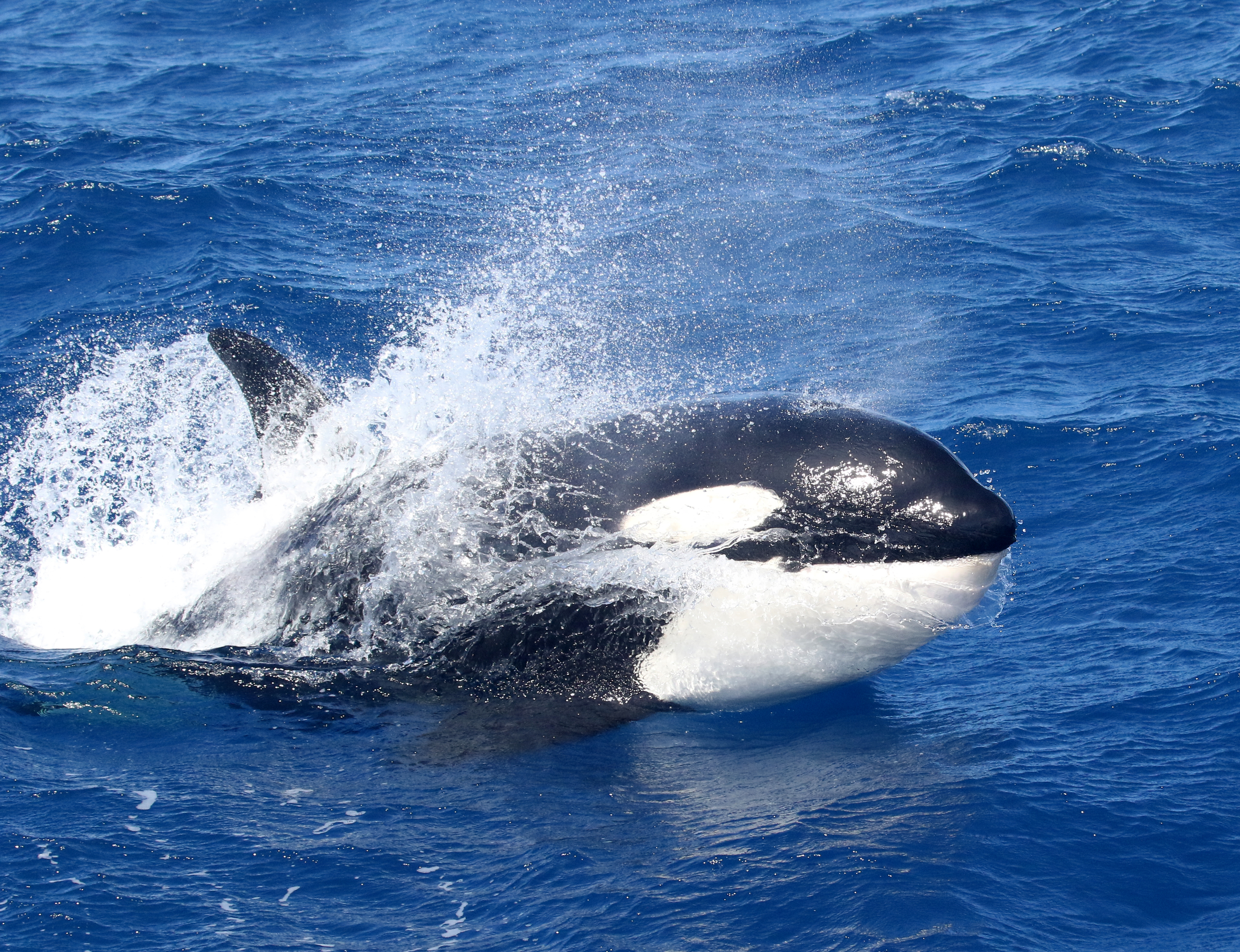

Supplement: Supplementary file 4 — 10.1186/s12898-016-0090-z “This was taken during my most recent field season studying the killer whales (Orcinus orca) at Bremer Canyon, Western [Australia] for my PhD. We were with a group of approximately 7 individuals taking fin shots, when all of a sudden the group had a change of direction and picked up their speed. They were on the hunt! We observed them charging and porpoising through the water at high speed chasing down their next meal. What ensued was a successful predation on another cetacean. Seeing them in all their might and glory and speed, you certainly gain respect for this King of the Ocean.” Attribution: Rebecca Wellard (Curtin University, Western Australia). [file 12898_2016_90_MOESM4_ESM.jpg]

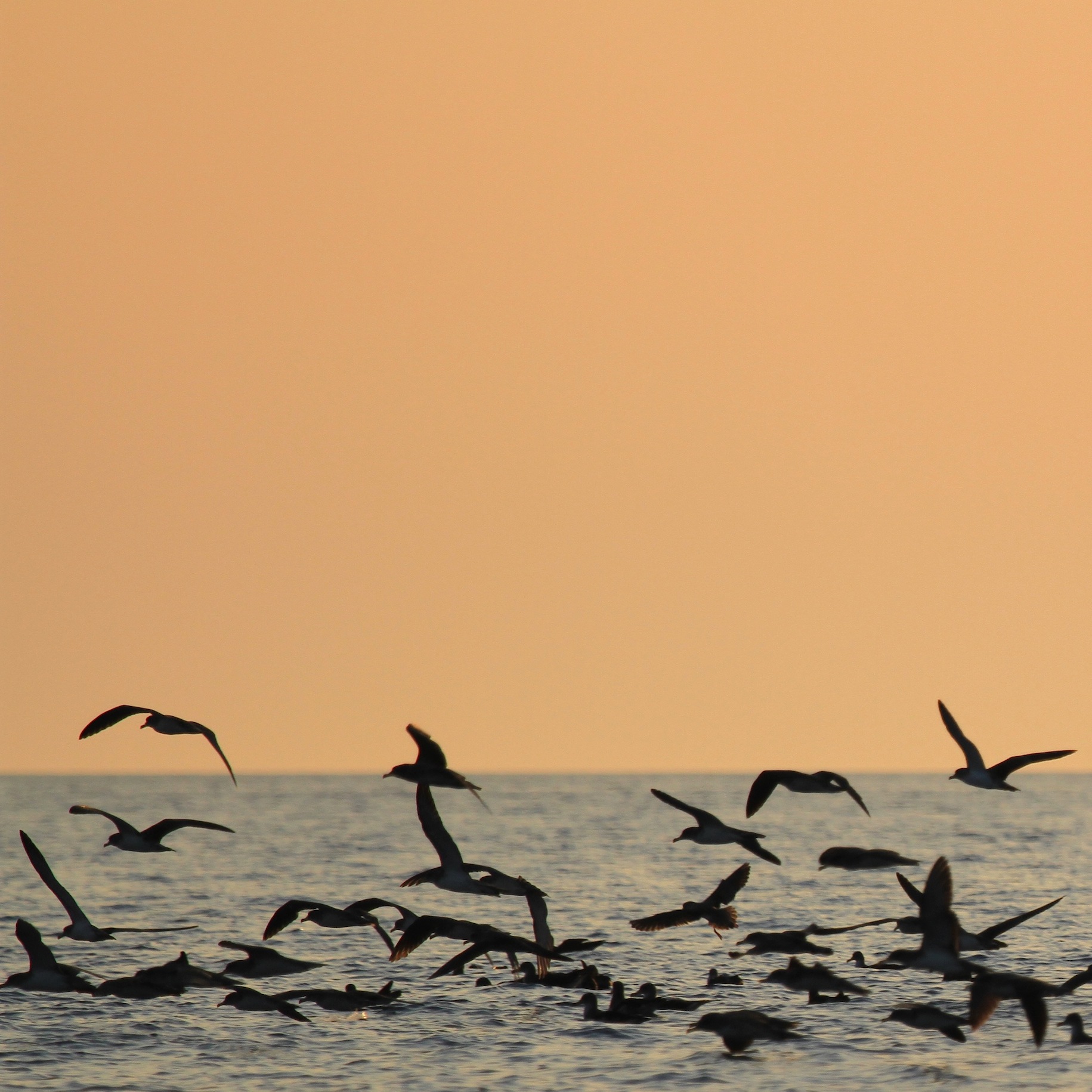

Supplement: Supplementary file 5 — 10.1186/s12898-016-0090-z “Linosa Island in Italy holds the second largest colony of Scopoli’s shearwaters (Calonectris diomedea) in the Mediterranean. Every day, before the sunset, shearwaters gather in large groups a few (kilometers) off shore, rafting on the sea patiently waiting for night when they can fly ashore.” Attribution: David Costantini (University of Antwerp, Belgium). [file 12898_2016_90_MOESM5_ESM.jpg]

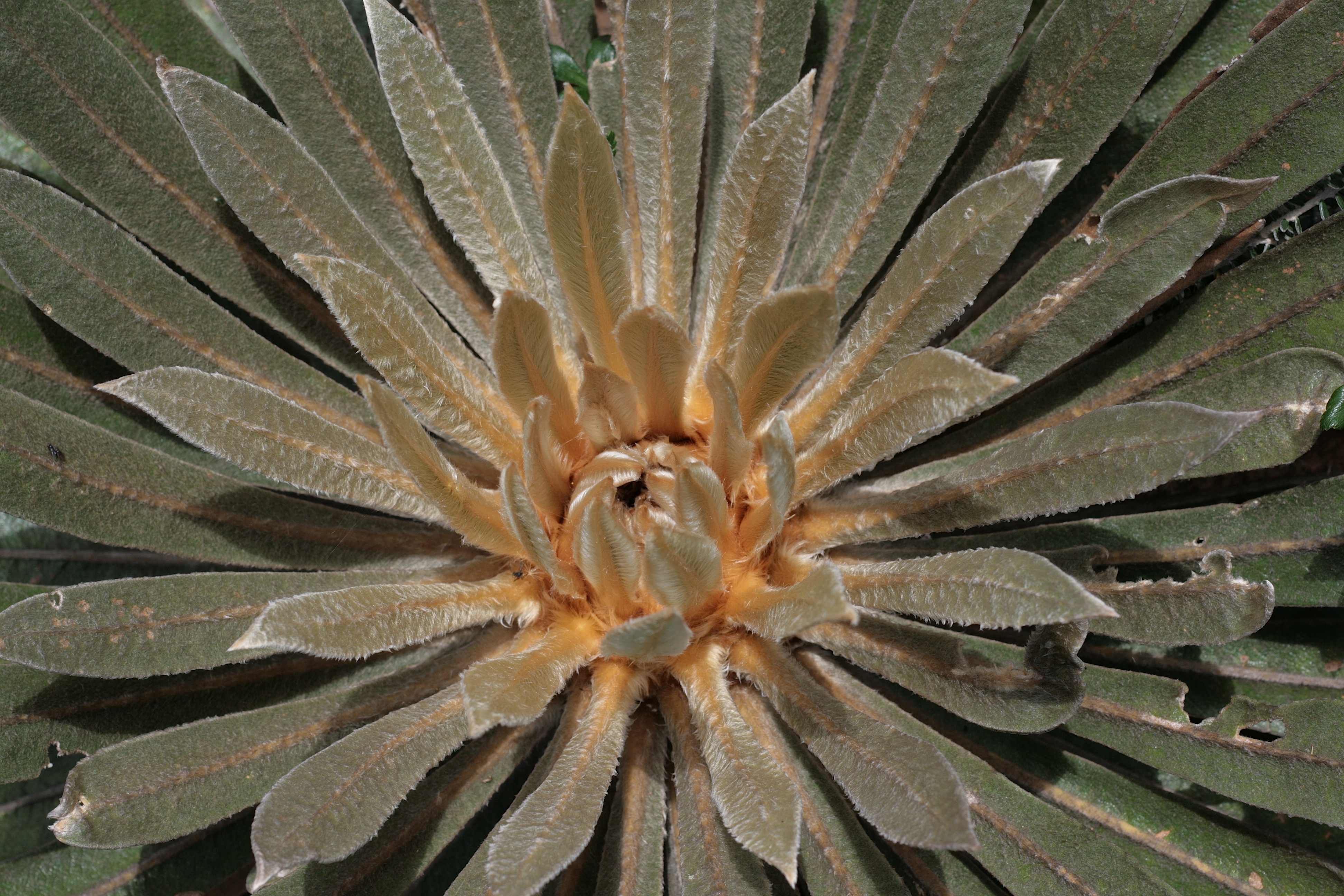

Supplement: Supplementary file 6 — 10.1186/s12898-016-0090-z “This picture was taken in Colombia, at the “Cruz Verde” Páramo in February 2015. The species shown here (Espeletia sp.) commonly called “frailejón” is an important and common element of the Páramo ecosystem. Their succulent leaves and thick hair layer are adaptations to cold and extreme temperatures characteristic of Páramos. Currently, the genus Espeletia comprises several species, some of them are highly threatened by climate change and human activities such as agriculture.” Attribution: Francisco Javier Velásquez Puentes (University of Gothenburg, Sweden). [file 12898_2016_90_MOESM6_ESM.jpg]

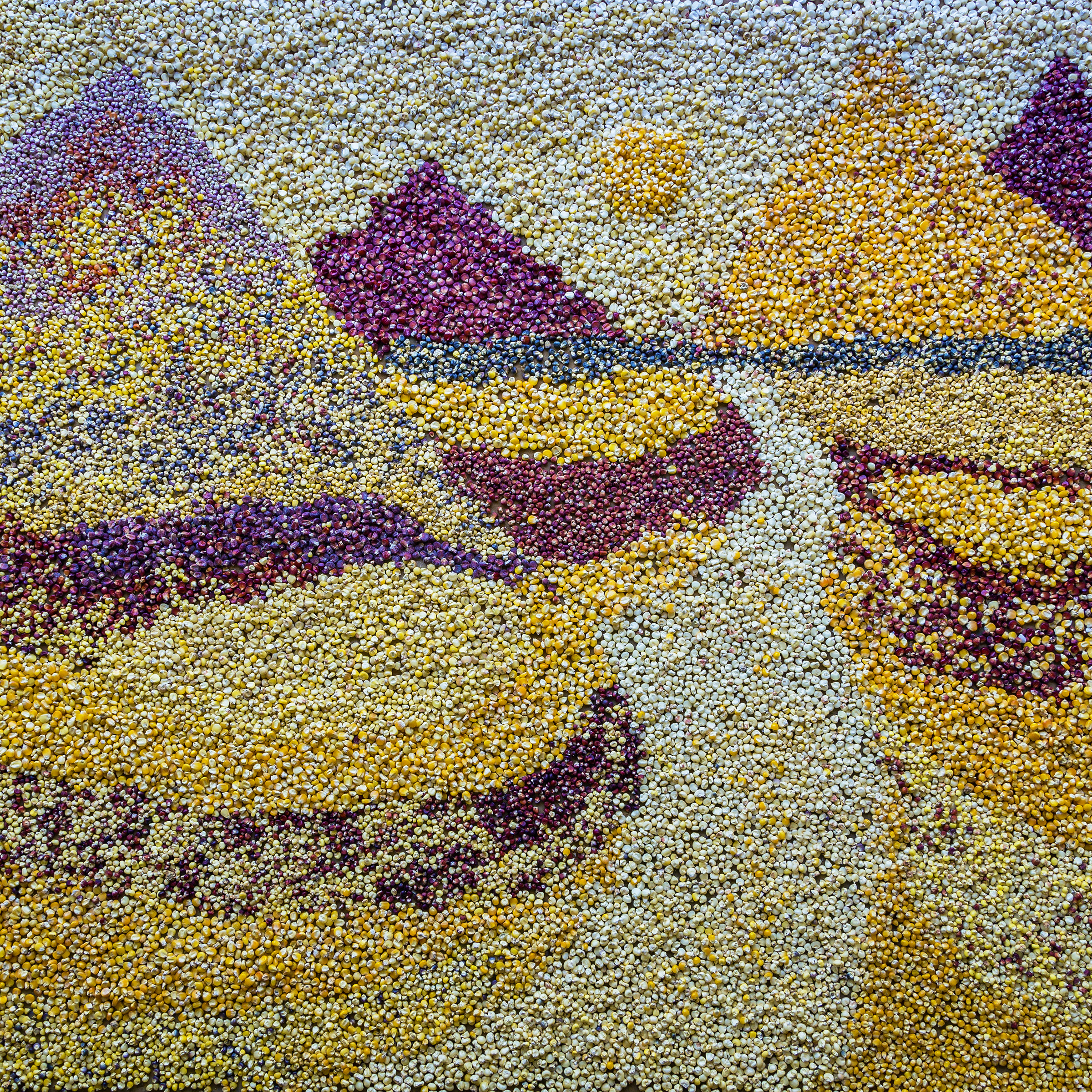

Supplement: Supplementary file 7 — 10.1186/s12898-016-0090-z “Maize (Zea mays) is the second most important crop of northeastern (NE) region of India. It [may] be considered as a secondary center of origin after Mexico. Occurrence of Sikkim primitive -1, a primitive and highly prolific maize landrace, in this region supports this theory. Many studies have shown that [diversity] of maize landraces in NE India is huge. However, a comparison with the global collections is required. [NE] India is dominated by (around 97 % of the total population) tribal communities. The soil and environmental heterogeneity of this region is also huge. Altogether, the ethnolinguistics and environment of NE India [led] to development [of] plant diversity. Around 5000 germplasm accessions of maize [have] already been collected and conserved from eight states of NE India. All eight states have maize resources, however the diversity is considerably high in Sikkim, Arunachal Pradesh, Nagaland, Manipur and Mizoram. Among the many, notable variation [has been] observed for [traits] such as cob size and shape, color of the kernels, plant architecture, amylose and protein content in the kernels, prolificacy, and more. The indigenous farms still rely on the traditional composite landraces for their food security. This photo represents the diversity in kernel morphology of some maize landraces conserved in ex situ gene bank of ICAR-National Bureau of Plant Genetic Resources, Regional Station, Umiam, Meghalaya, India.” Attribution: Somnath Roy (Indian Council of Agricultural Research, India). [file 12898_2016_90_MOESM7_ESM.jpg]

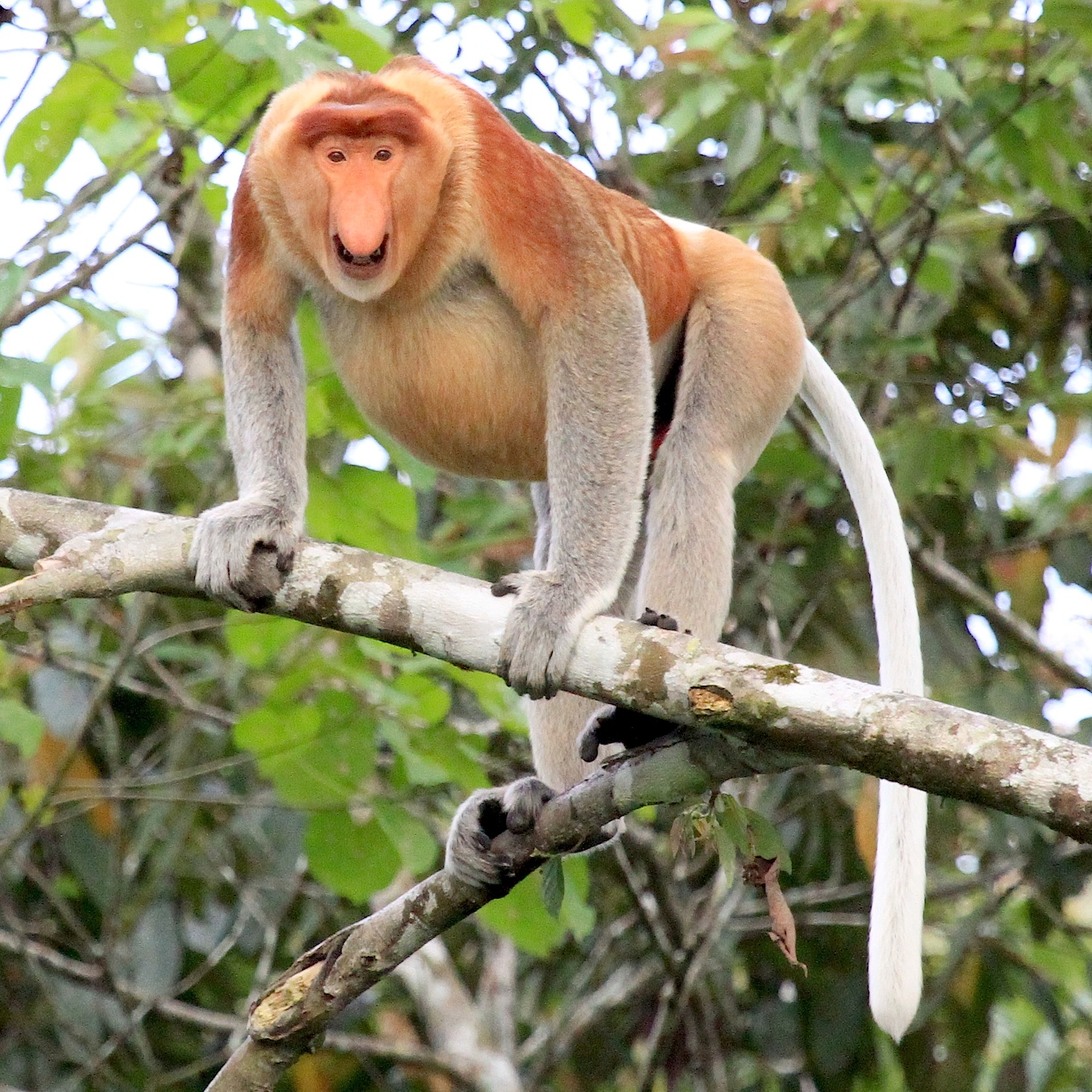

Supplement: Supplementary file 8 — 10.1186/s12898-016-0090-z “Dominant males of the proboscis monkey (Nasalis larvatus) found only on the island of Borneo defend strenuously their females against intruders.” Attribution: David Costantini (University of Antwerp, Belgium). [file 12898_2016_90_MOESM8_ESM.jpg]

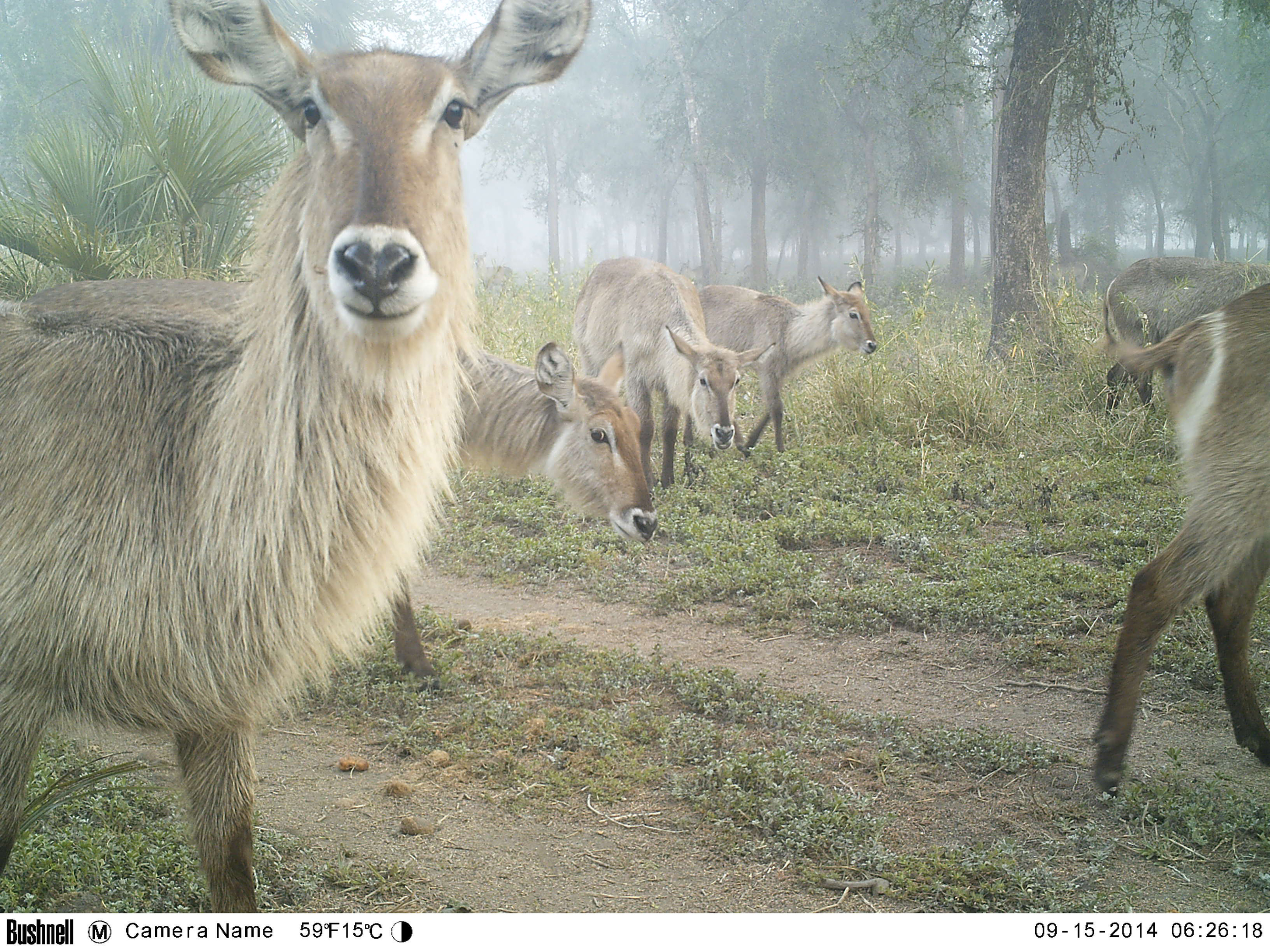

Supplement: Supplementary file 9 — 10.1186/s12898-016-0090-z “This photo of a herd of waterbuck in the morning mist was taken by a motion-detecting trail camera in the Gorongosa National Park in Mozambique. A network of fifty trail cameras were set up by Paola Bouley, a researcher who is studying how the lion population is rebounding in Gorongosa after decades of war devastated wildlife populations. Hundreds of thousands of photos that she and her team have collected are available for citizen scientists to help her identify on the website WildCam Gorongosa. Waterbuck are a common sight in Gorongosa as their population has exploded to over 34,000 individuals up from only a few hundred after the war. Scientists are studying the waterbuck population to learn why they are experiencing such rapid growth.” Attribution: Chuck Schultz (Science Education Department, Howard Hughes Medical Institute). [file 12898_2016_90_MOESM9_ESM.jpg]

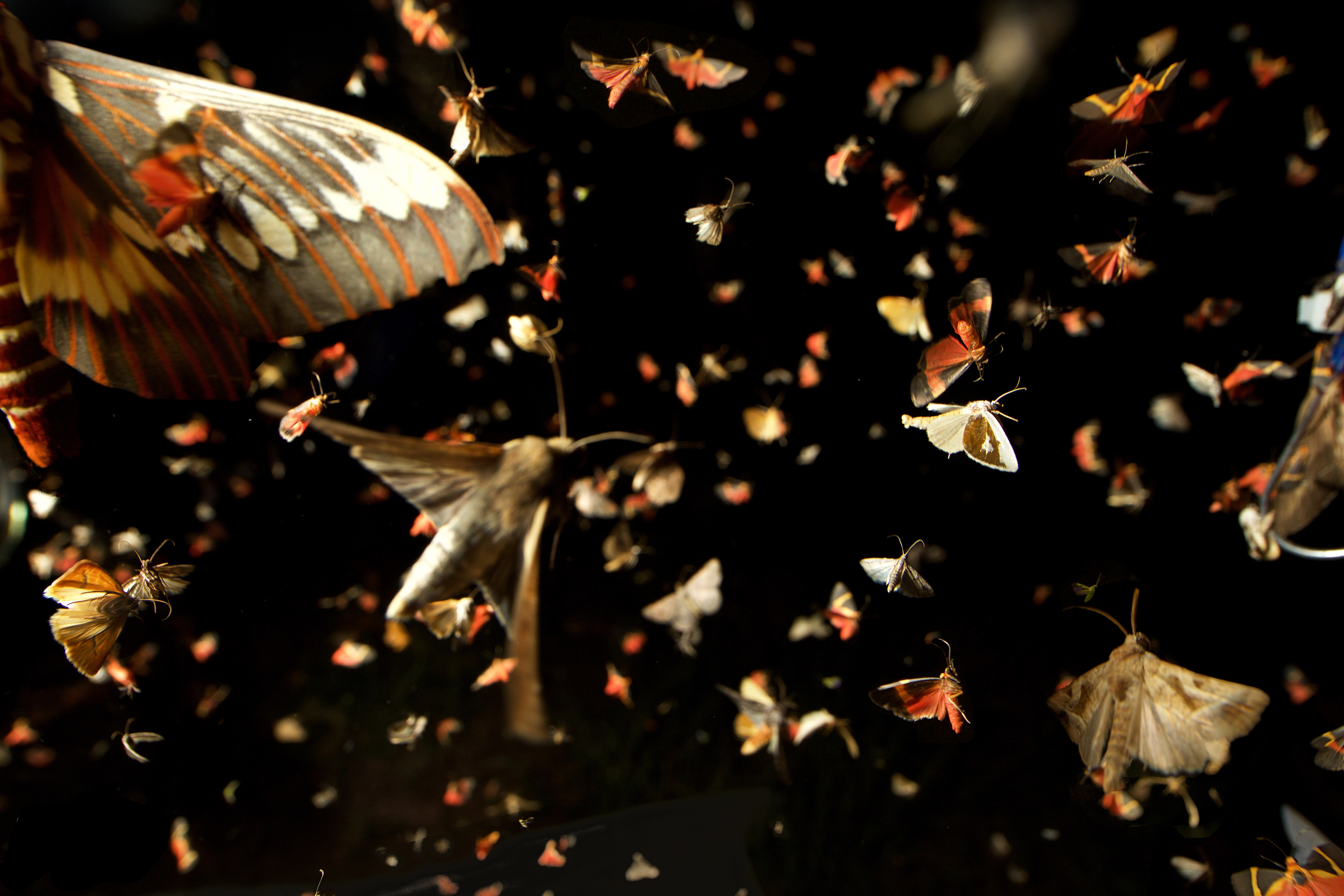

Supplement: Supplementary file 10 — 10.1186/s12898-016-0090-z “Every year, the monsoon rains of late summer bring the Sonoran Desert to life. Most of the region’s insect species time the emergence of adult life stages with the arrival of the monsoons. Entomologists often use lights as lures for insects. Light trapping in southeastern Arizona during this time of year attracts insect numbers on par with the tropics. A typical light trap consists of a white bed sheet hung perpendicular to the ground, with a bright light in front of it. At the end of the night, when the lights go off, we used a dim UV light to attract the swarms of moths and other insects so they don’t dive bomb our head lamps, or fill the open doors of our vehicles. Using a dim light to attract the moths from the sheet keeps them out of trouble and distracted, so that the equipment can be packed. This photo was taken at the end of a night of collecting, as the moths swarmed the UV light.” Attribution: Lawrence Reeves (University of Florida). [file 12898_2016_90_MOESM10_ESM.jpg]

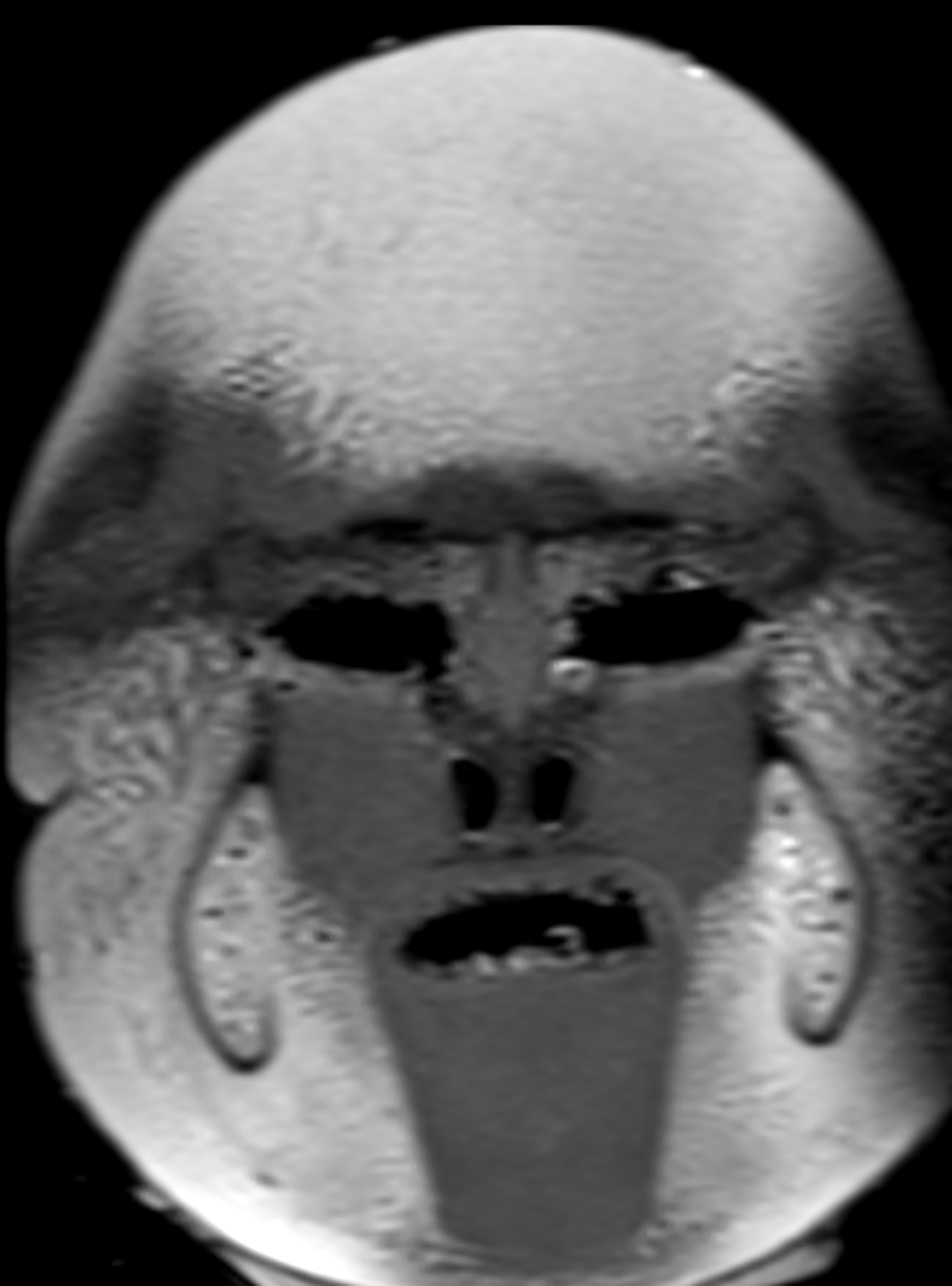

Supplement: Supplementary file 11 — 10.1186/s12898-016-0090-z “This picture was taken in the frame of a pluridisciplinary study of the biochemical and anatomical basis of echolocation functioning in toothed whales. As a part of this study, specialists of medical imaging of the Brest Hospital (France) use MRI (Magnetic Resonance Imaging) to decipher the complex anatomical structure of “acoustic fat” tissues (melon and mandibular fats) in odontocetes. It [was] during such a working session (grouping Marion Arribart, veterinary student at ONIRIS, Nantes; Douraied Ben Salem and Julien Ognard, medical doctors, specialists of medical imaging, CHRU of Brest; and Jean-Luc Jung, molecular biologist and geneticist, BioGeMME laboratory, University of Brest), that Douraied Ben Salem had his attention drawn by this strange and surprising image of a harbor porpoise head, appearing on the screen, among other more classical pictures. The MRI captured the harbor porpoise head as an amazing kind of tribal mask, where the melon and the mandibular fats are visible, inter-bones spaces and the pharynx appear as eyes and mouth, and mandibulae look like very visible earrings, thus giving a strange artistic dimension to this scientific picture. The picture has not been modified or altered in any way.” Attribution: Jean-Luc Jung (University of Brest, CHRU of Brest, ONIRIS). [file 12898_2016_90_MOESM11_ESM.tif]

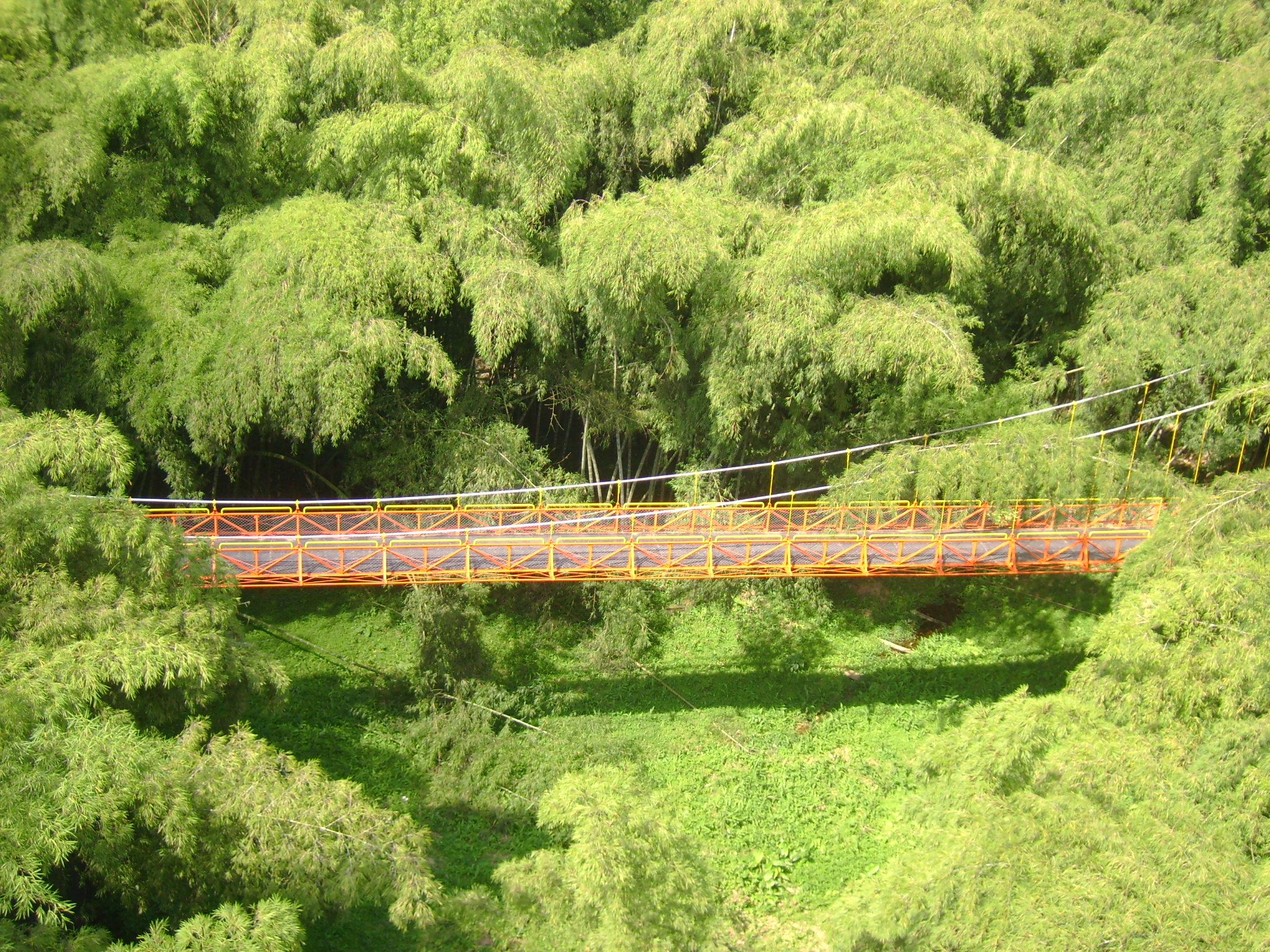

Supplement: Supplementary file 12 — 10.1186/s12898-016-0090-z “Bamboo plants in perfect harmony with buildings of interest to human communities. Prevailing respect for nature and its species.” Attribution: Arnubio Valencia Jimenez (University of Caldas, Colombia). [file 12898_2016_90_MOESM12_ESM.jpg]

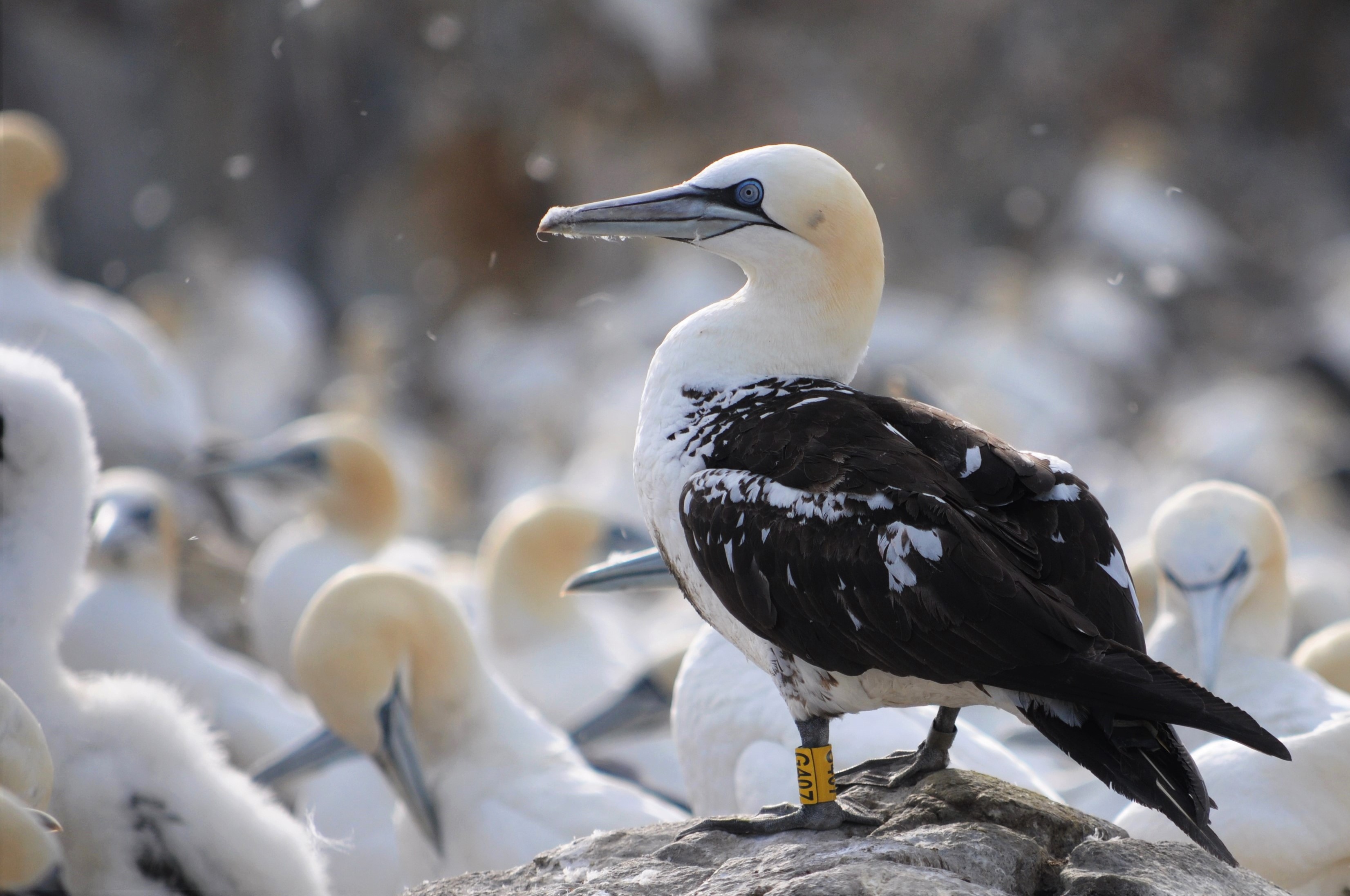

Supplement: Supplementary file 13 — 10.1186/s12898-016-0090-z “G407 is an immature gannet that was fitted with a unique colored band and a GPS logger, which is just visible on the tail. Steve Votier and I tagged this bird as a part of project run by University of Glasgow’s Jana Jeglinski, which investigates the movements of gannets before they settle down to breed, as very little is known about this 4–5 year period. These solar-powered loggers ‘text’ GPS data via the mobile phone network, so the bird can be tracked in real time until the bird naturally shed the tag as she replaced her tail feathers. After leaving RSPB Grassholm Island in West Wales, a colony of 36,000 breeding pairs, she travelled to Ireland, Cornwall and France!” Attribution: Bethany Clark (University of Exeter, UK). [file 12898_2016_90_MOESM13_ESM.jpg]

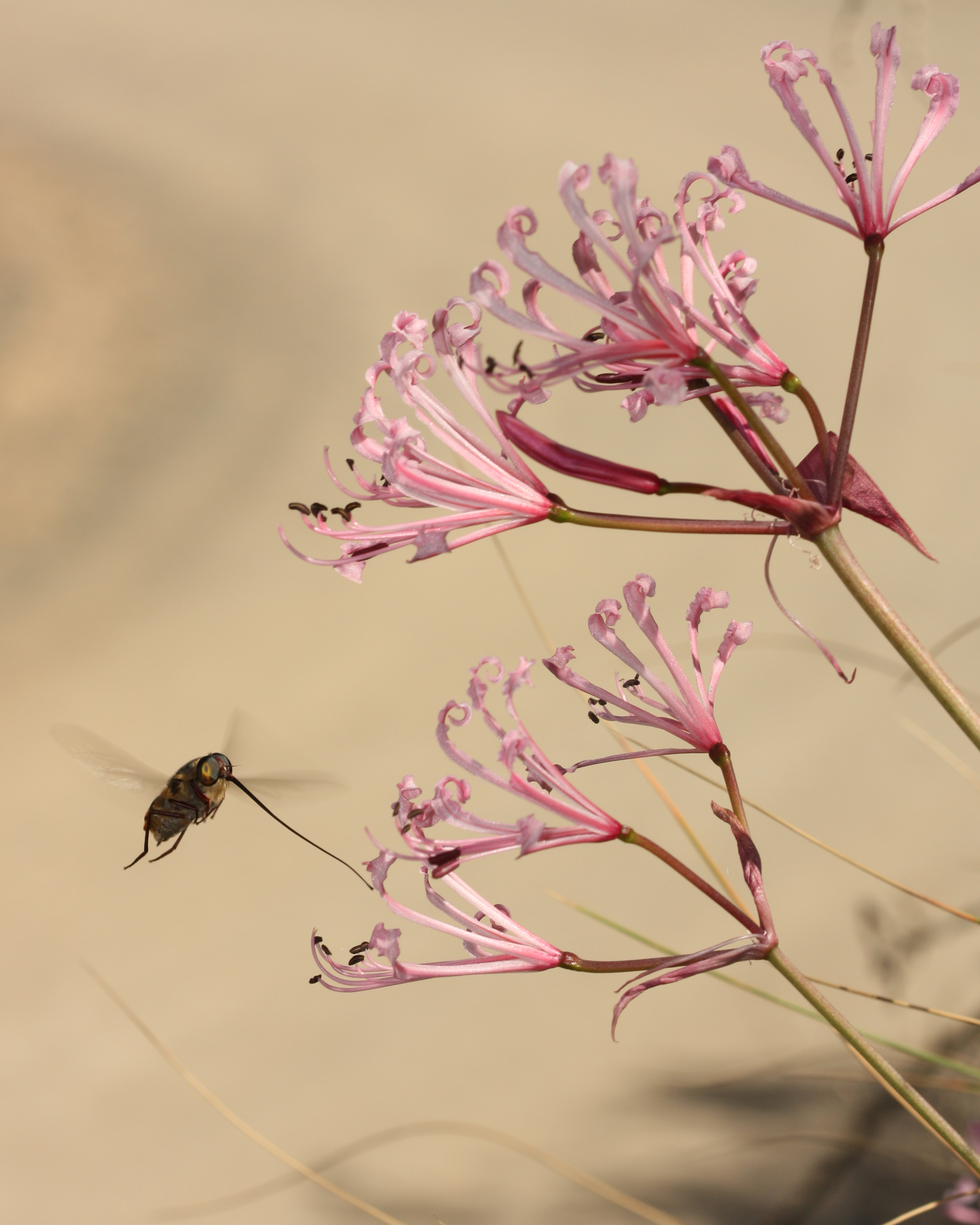

Supplement: Supplementary file 14 — 10.1186/s12898-016-0090-z “The long proboscid fly, Prosoeca longipennis approaches the inflorescences of Nerine humilis (Amaryllidaceae). The distribution of the flies is confined to just a few N. humilis populations in South Africa, and the unusually long styles and anthers of those N. humilis populations are locally adapted to receive and deposit pollen on the abdomens of the flies.” Attribution: Ethan Newman (University of Stellenbosch, South Africa). [file 12898_2016_90_MOESM14_ESM.jpg]

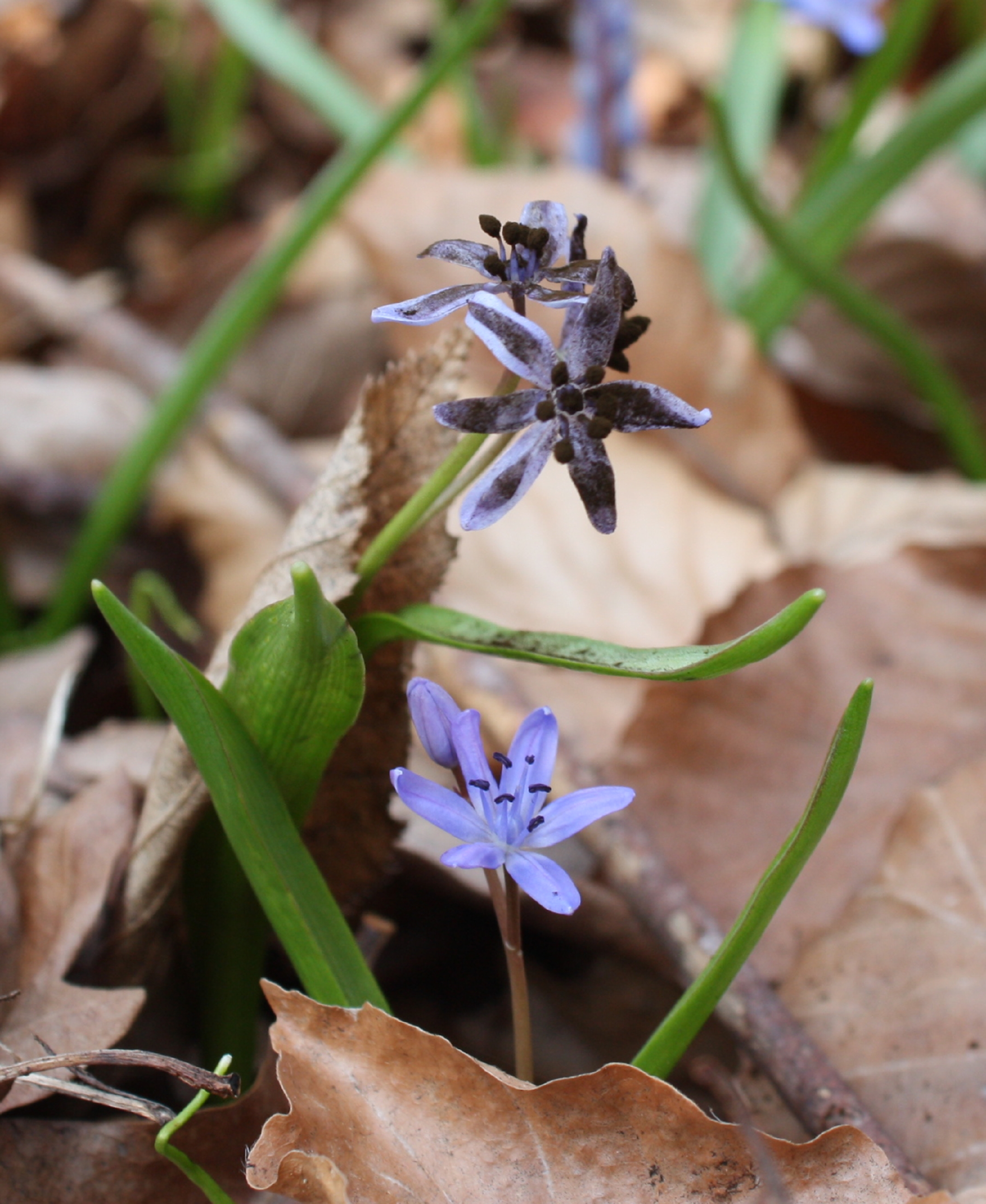

Supplement: Supplementary file 15 — 10.1186/s12898-016-0090-z “Beauty is in the eye of the beholder: Antherospora scillae on Scilla bifolia in an early spring beech grove. Most people rejoice in the beauty of early bloomers like squills or snowdrops heralding spring. For parasitologists delight is even bigger if plants are infected by organisms that live at the expense of their host. The Antherospora smut fungi sporulate in the anthers and on the surface of the inner floral organs of different Hyacinthaceae replacing the pollen and thus sterilizing the host plant. Quite recently they were shown to represent a distinct phylogenetic lineage that now includes 12 species some of which are morphologically similar but phylogenetically different and strictly host species specific. The mind of non-parasitologists may be put at ease: Hudson et al. (2006) considered a healthy system to be one that is rich in parasite species!” Attribution: Matthias Lutz (University of Tübingen, Germany). [file 12898_2016_90_MOESM15_ESM.jpg]

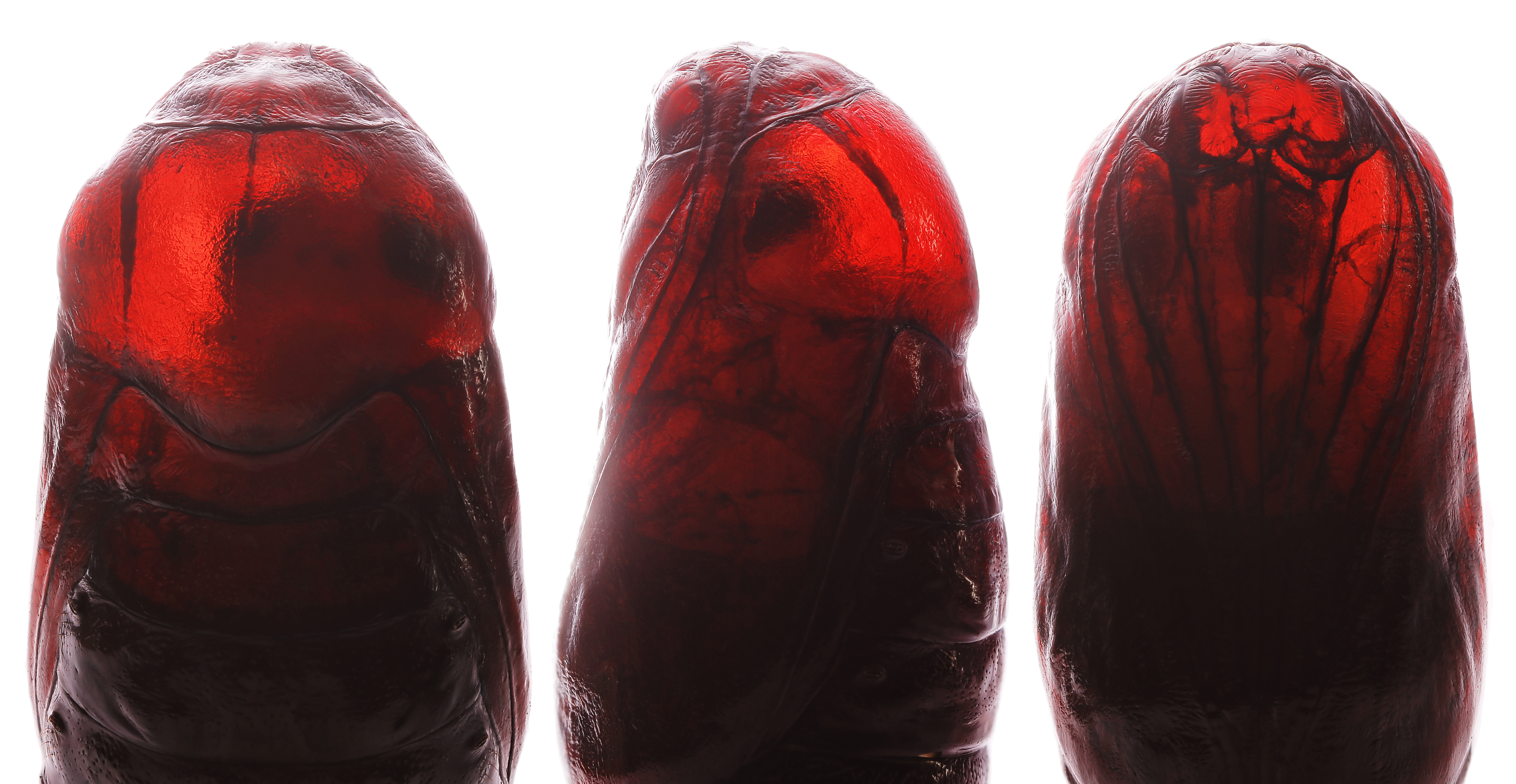

Supplement: Supplementary file 16 — 10.1186/s12898-016-0090-z “Metamorphosis of a parasitoid in its host. Typically, it is the developing pair of compound eyes that, due to their dark pigmentation, are first distinguishable in a moth pupa during its metamorphosis. Sometimes, however, these eyes do not belong to the moth but to a parasitoid wasp. In the image presented, it is the pupa of Heteropelma amictum (Hymenoptera: Ichneumonidae), that is developing in its host, Callimorpha dominula (Lepidoptera: Erebidae). Attribution: Franziska Bauer (Senckenberg Natural History Collections Dresden, Germany). [file 12898_2016_90_MOESM16_ESM.jpg]

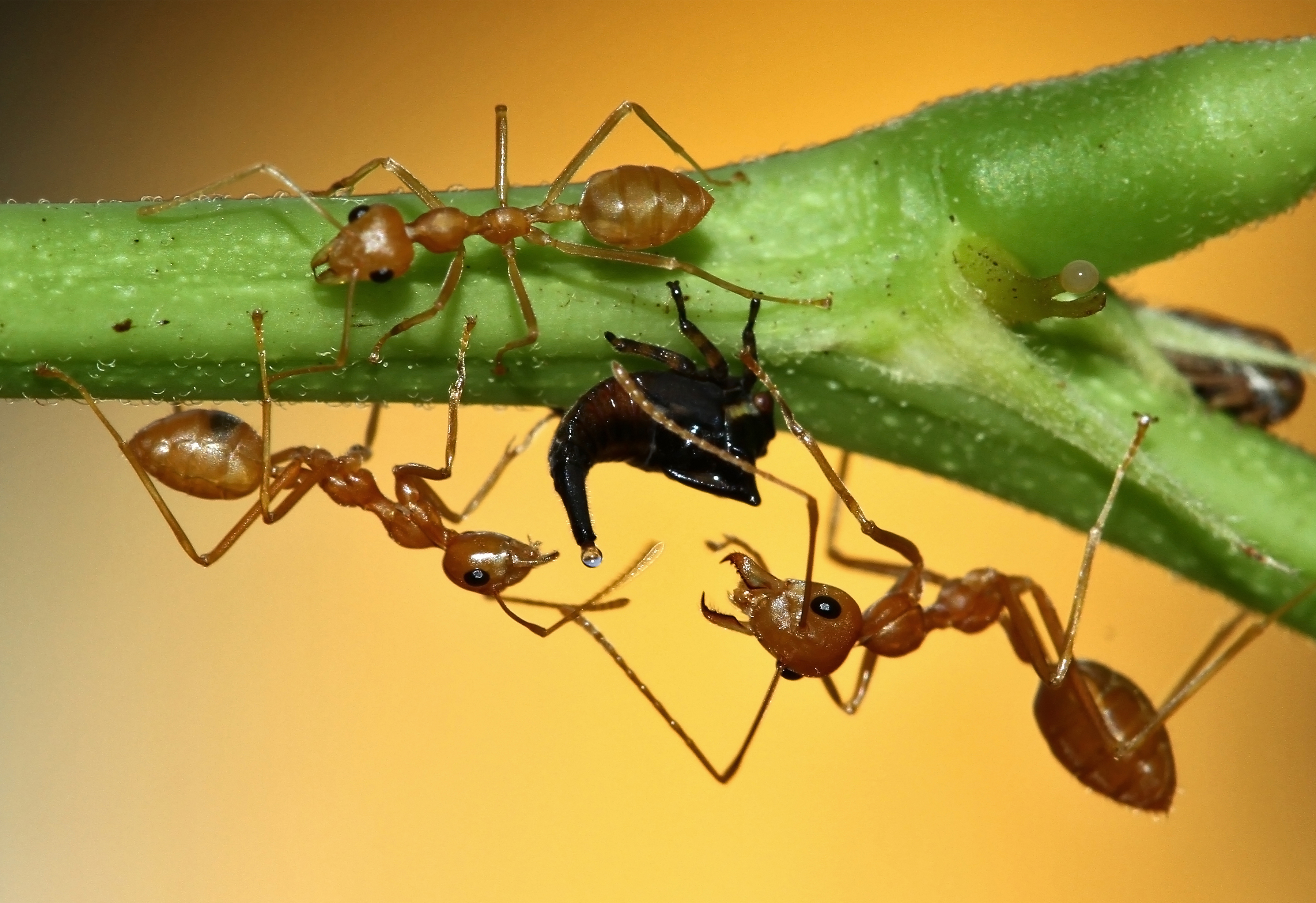

Supplement: Supplementary file 17 — 10.1186/s12898-016-0090-z “Myrmecophily is a well-known mutualistic interaction between ants and homopterans. Weaver ants (Oecophylla smaragdina) are behaviorally very dominant species of ants, and they generally predate upon other insects. During my 3 years [of] field work in a mixed deciduous forest in India, I found Oecophylla ants to mostly have protein diet & using baiting experiment[s], I found them to prefer protein baits over honey baits. Also I didn’t find any Oecophylla individual tending aphids in the forest. In my garden, however, I found Oecophylla ants to tend homoptera nymphs (depicted here) on a regular basis. Perhaps the availability of protein foods is an important regulating factor of their homoptera rearing behavior. I also found the ants carrying the hopper nymphs with their mandibles and placing them at different parts of the plant.” Attribution: Arpan Kumar Parui (University of Calcutta, India). [file 12898_2016_90_MOESM17_ESM.jpg]

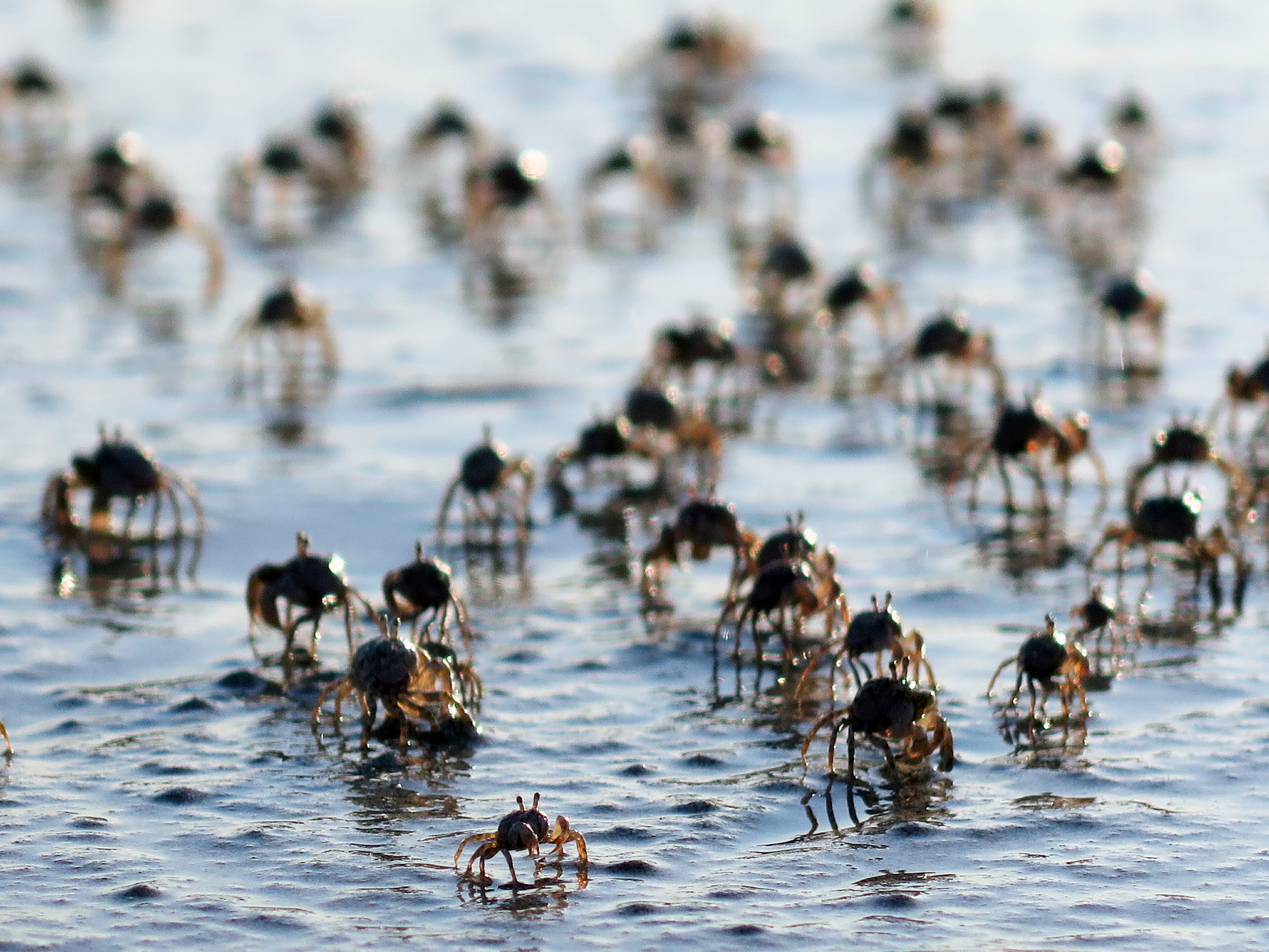

Supplement: Supplementary file 18 — 10.1186/s12898-016-0090-z “Sand bubbler crabs live on soft shores of the tropical Indo-Pacific. They live in burrows in the sand, where they hide during high tide. During low tide, while searching the sand for food, they form characteristic sand pellets which cover the sand. They play an important ecological role as deposit feeders and bioturbators, and have been shown to affect the productivity of sandy shores. They move in large groups and scout the beach radially from their burrows, creating intricate and characteristic patterns of pellets as they proceed.” Attribution: Ulisse Cardini (University of Vienna, Austria). [file 12898_2016_90_MOESM18_ESM.jpg]
